# Comparative Analysis of the miRNome of Bovine Milk Fat, Whey and Cells

**DOI:** 10.1371/journal.pone.0154129

**Published:** 2016-04-21

**Authors:** Ran Li, Pier-Luc Dudemaine, Xin Zhao, Chuzhao Lei, Eveline Mengwi Ibeagha-Awemu

**Affiliations:** 1 College of Animal Science and Technology, Northwest A & F University, Xi’an, Shaanxi, 712100, China; 2 Agriculture and Agri-Food Canada, Sherbrooke Research and Development Centre, Sherbrooke, Quebec, J1M 0C8, Canada; 3 Department of Animal Science, McGill University, 21111, Lakeshore Road, Ste-Anne-de Bellevue, Quebec, J1M 0C8, Canada; University of Tennessee Health Science Center, UNITED STATES

## Abstract

Abundant miRNAs have been identified in milk and mammary gland tissues of different species. Typically, RNA in milk can be extracted from different fractions including fat, whey and cells and the mRNA transcriptome of milk could serve as an indicator of the transcriptome of mammary gland tissue. However, it has not been adequately validated if the miRNA transcriptome of any milk fraction could be representative of that of mammary gland tissue. The objectives of this study were to (1) characterize the miRNA expression spectra from three milk fractions- fat, whey and cells; (2) compare miRNome profiles of milk fractions (fat, whey and cells) with mammary gland tissue miRNome, and (3) determine which milk fraction miRNome profile could be a better representative of the miRNome profile of mammary gland tissue. Milk from four healthy Canadian Holstein cows in mid lactation was collected and fractionated. Total RNA extracted from each fraction was used for library preparation followed by small RNA sequencing. In addition, miRNA transcripts of mammary gland tissues from twelve Holstein cows in our previous study were used to compare our data. We identified 210, 200 and 249 known miRNAs from milk fat, whey and cells, respectively, with 188 universally expressed in the three fractions. In addition, 33, 31 and 36 novel miRNAs from milk fat, whey and cells were identified, with 28 common in the three fractions. Among 20 most highly expressed miRNAs in each fraction, 14 were expressed in common and 11 were further shared with mammary gland tissue. The three milk fractions demonstrated a clear separation from each other using a hierarchical cluster analysis with milk fat and whey being most closely related. The miRNome correlation between milk fat and mammary gland tissue (r_mean_ = 0.866) was significantly higher than the other two pairs (p < 0.01), whey/mammary gland tissue (r_mean_ = 0.755) and milk cell/mammary gland tissue (r_mean_ = 0.75), suggesting that milk fat could be an alternative non-invasive source of RNA in assessing miRNA activities in bovine mammary gland. Predicted target genes (1802) of 14 highly expressed miRNAs in milk fractions were enriched in fundamental cellular functions, infection, organ and tissue development. Furthermore, some miRNAs were highly enriched (FDR <0.05) in milk whey (3), cells (11) and mammary gland tissue (14) suggesting specific regulatory functions in the various fractions. In conclusion, we have obtained a comprehensive miRNA profile of the different milk fractions using high throughput sequencing. Our comparative analysis showed that miRNAs from milk fat accurately portrayed the miRNome of mammary gland tissue. Functional annotation of the top expressed miRNAs in milk confirmed their critical regulatory roles in mammary gland functions and potentially to milk recipients.

## Introduction

Cow milk is produced to promote the growth and developmental needs of young calves by nature as is for other mammalian species. Cow milk is a good resource of numerous essential nutrients including proteins, lipids, and amino acids as well as other bioactive components including hormones and cytokines. Due to its nutritious significance, cow milk has been commercialized and routinely consumed by humans for growth and health benefits. In addition to these nutritional components, milk from cows [1–4] and other species [5–9] are also rich in microRNAs (miRNAs) which play important roles in posttranscriptional regulation of gene expression [10].

Milk can be fractionated into three parts including fat, whey and somatic cells through low and high speed centrifugations [11–13]. Low speed centrifugation will separate milk into three visible layers including a fat layer (mainly fat globules) at the top, a middle fluid phase and cell pellets at the bottom. Milk fat globules are secreted by mammary epithelial cells (MECs) via a budding mechanism which envelopes a crescent of the MEC cytoplasm in plasma membrane [14]. With further high speed centrifugation and microfiltration, the fat residues and protein micelles in the fluid phase can be removed, resulting in a homogenous whey phase. This defatted and cell-free whey fraction contains exosomes, which are secreted into milk by MECs in the form of small (10–100 nm diameter) membrane vesicles containing mRNA and miRNA [2,8,11]. The milk cells are heterogeneous, predominated by leucocytes with a small proportion of exfoliated mammary epithelial cells.

MiRNA from milk whey exist mainly in the exosomes which can prevent miRNA from degradation under harsh conditions of low pH and RNase treatment [3]. To date, a number of studies have explored the miRNome of milk whey fraction with a large number of whey miRNAs identified in cattle [11,15], pig [7], rat [8], wallaby [9] and human [6]. Additionally, six miRNAs in milk exosomes were found to be differentially expressed in response to bacterial infection of bovine mammary gland [16]. MiRNAs are also present in milk fat globules of humans [12] and bovine [17], and have been profiled using next generation sequencing technology [18]. Although miRNAs are found to be expressed in human somatic cells [12], the overall miRNA spectrum in milk somatic cells of cattle and other farm animal species remains unclear.

MiRNA expression in bovine milk is not merely for the benefit of mammary gland processes/functions. Compelling evidence has shown that milk derived miRNAs may have potential regulatory roles in modulating the immune system or metabolic processes of milk recipients [1,19–21]. Studies have shown that miRNAs could be absorbed by humans in biologically meaningful amounts which could affect related gene expression in peripheral blood mononuclear cells [22]. Another study has further confirmed that whey exosomes containing miRNAs and mRNA could be absorbed by human macrophages [15], implying a possible function of these miRNAs in the human body. In addition, milk miRNAs can be resistant enough to be detected in raw and commercial milk and other dairy products [23], in spite of losses during processing and storage [24]. Considering the high consumption of bovine milk and dairy products by humans, a comprehensive study of the miRNome profile of milk is a critical step towards investigation of the regulatory roles of cow miRNAs in humans.

The understanding of the miRNome profile of the different milk fractions will also help to determine which milk fraction could be used as a source of RNA for the study of mammary gland functions. Sampling of mammary gland tissue through the biopsy approach has been the standard source of RNA used to investigate the transcriptome activities of lactating mammary epithelial cells. However, the biopsy approach to collect mammary gland tissue is costly, invasive, and usually leads to infection of the mammary gland, which prevents a repeat sampling as required by many experiments. At the mRNA expression level, studies have found that RNA from milk fat globules and somatic cells are good representation of mammary gland tissue [25–28] and thus can be used as a non-invasive source of mRNA for the study of mammary gland biology. However, miRNAs are small regulatory molecules (around 22nt) which are much shorter than mRNA and have distinct biogenesis from mRNA [29]. So far, it has not been systematically verified whether the miRNome of milk fat globules or somatic cells could serve as a good representation of that of mammary gland tissue. Besides, no study has compared the similarity of milk whey, fat and somatic cells transcriptomes with that of mammary gland tissues at the miRNA level. Therefore, a comprehensive comparison of the miRNome profile of the different milk fractions would help to determine which fraction could best represent the miRNome profile of mammary gland tissues. Additionally, knowledge of the miRNome profile of the different fractions of milk and mammary gland tissue will aid in informed decisions in choosing a particular milk fraction as a non-invasive source of miRNA to answer specific questions regarding mammary gland biology.

In order to obtain a comprehensive profile of milk miRNAs, we examined the miRNome of milk fat globules, whey and somatic cells of the same cows, and compared with that of mammary gland tissues. Our study would determine the best alternative and non-invasive sampling method to study the miRNA expression in bovine mammary gland. Furthermore, a comprehensive discovery of miRNAs in milk would be of great value for exploring their regulatory functions in further studies.

## Materials and Methods

### Ethics statement

All the experimental procedures were according to the national codes of practice for the care and handling of farm animals (http://www.nfacc.ca/codes-of-practice) and approved by the Animal Care and Ethics Committee of Agriculture and Agri-Food Canada. Animals were cared for following standard management procedures and were allowed ad libitum access to feed and water. Cows were fed with a diet consisting of a total mixed ration of corn and grass silages (50:50) and concentrates.

### Milk collection and fractionation

Four healthy Canadian Holstein cows in mid lactation (130–160 days in milk) were chosen for milk collection. Fresh milk samples were collected three hours after the morning milking. A volume of 50 mL milk was collected from the back quarters of each cow and immediately placed on ice, transferred to laboratory and processed to reduce potential RNA degradation.

Milk was mixed well before centrifugation at 1,900g for 15 min. The fat in the upper phase, whey in the middle phase as well as the cells at the bottom of the tube were each transferred to a new 50 mL RNase free falcon tube. Each fraction was homogenized before RNA isolation following different methods. About 7.5 mL Qiazol lysis reagent (Qiagen Inc., USA) was added to the fat, vigorously mixed by vortexing until the fat was well dispersed. Milk cells were washed twice with cold PBS and then homogenized with 1 mL of Qiazol lysis reagent. Milk whey was homogenized following a protocol by Izumi [11] with modifications. Briefly, milk whey was centrifuged twice at 21,500 g for 1 h at 4°C to remove caseins and residual fat. The clear whey supernatant was passed through 0.80, 0.45 and 0.22 μm (in that order) filters (Sterlitech Corporation, USA) to remove residual cell debris. In order to increase the yield of whey RNA, whey samples were lyophilized. Two 5 mL aliquots of each whey sample were each placed in a 50 mL RNase free falcon tube and lyophilized for 3 hours at 0°C followed by 10 hours at 4°C using a Virtis Genesis 25XL Lyophilizer (SP Scientific, USA). Finally, the lyophilized milk whey was homogenized with Qiazol lysis solution (1:2, e.g. 5 mL Qiazol lysis solution: 10 mL milk whey). All the homogenates (milk fat, cells and whey) were stored at -80°C until used.

### Total RNA extraction

Total RNA was extracted using miRVana miRNA isolation kit (Life Technologies, USA) following manufacturer’s instructions. Briefly, 1/10 volume of miRNA Homogenate Additive was added respectively to homogenates from the fractionation step (5 mL fat homogenate, 5 mL whey homogenate and 1 mL cell homogenate) and mixed well. Then, one volume of acid-phenol:chloroform (equal to the homogenate before adding miRNA Homogenate Additive) was added to the homogenate and thoroughly mixed. The resulting aqueous phase was mixed with 1.25 volumes of room temperature 100% ethanol and then passed through a filter cartridge (Life Technologies Corporation, USA). Next, the filter cartridge was washed with the supplied wash solution before eluting RNA with pre-heated (95°C) elution solution. RNA was then digested with Turbo DNase (Ambion Inc., USA) to remove genomic DNA contaminant. Finally, the digested RNA was purified using Zymo RNA clean & concentrator-25 (Zymo Research, USA). The quantity of RNA was measured using NanoDrop 1000 (NanoDrop Technologies, USA). RNA integrity was further determined on an Agilent 2100 Bioanalyzer using an RNA 6000 Pico kit (both from Agilent Technologies, USA).

### Library preparation and small RNA sequencing

Twelve libraries for the three fractions of milk (fat, cell and whey) of 4 cows were prepared and barcoded for sequencing according to Vigneault et al. [30] with minor modifications reported in Li et al.[17]. Briefly, total RNA was first ligated to a 3’ adaptor and then annealed to a reverse transcription primer to prevent undesirable dimerization of 3’ and 5’ adaptors in the following step. Before reverse transcription, the 5’ adaptor was ligated to the 5’end of the RNA. This RNA:DNA hybrid was then reversely transcribed into cDNA using a Superscript III kit (Life Technologies, USA). Each library was barcoded by PCR with a unique barcode in the reverse primer using NEBNext high-fidelity 2× PCR master mix (New England Biolabs, Canada). The PCR products corresponding to small RNA were selected using polyacrylamide gel electrophoresis. The concentration of the purified libraries was evaluated by a Picogreen assay (Life Technologies) on a Nanodrop 3300 fluorescent spectrophotometer (Thermo Scientific, USA).

The 12 libraries were multiplexed and subjected to 50bp single end sequencing on one lane using an Illumina HiSeq 2000 system (Illumina Inc., USA) by McGill University and Genome Quebec Innovation Centre (Montreal, QC, Canada). Raw fastq files of the sequence data have been submitted to NCBI Sequence Read Archive database with accession number SRX1603675.

### Small RNA sequencing data analysis

The fastq files (raw sequence data) were checked for sequencing quality with the FastQC program version 0.10.1 (http://www.bioinformatics.babraham.ac.uk/projects/fastqc/). The Cutadapt v1.2.2 (http://code.google.com/p/cutadapt/) program was used to trim 3’ adaptor sequences and to remove reads which were shorter than 18 nucleotides after trimming or had a low Phred score of less than 20 for at least 50% of the bases. Clean reads were mapped to the bovine genome (UMD3.1) using bowtie 1.0.0 [31]. Reads that mapped to more than five positions were discarded. Furthermore, reads that mapped to bovine rRNA, tRNA, snRNA and snoRNA in the Rfam RNA family database (http://rfam.sanger.ac.uk/) were also removed.

Identification of known miRNA and discovery of novel miRNA were performed using miRDeep2 v2.0.0.7 [32] with miRBase release 21 as reference. Reads from all the libraries were pooled together for novel miRNA prediction using the miRDeep2 core module which outputs a scored list of known and novel miRNAs with log-odds score to help determine false positives. In this study, only miRNAs with at least 10 CPM (count per million) in at least 2 libraries of any milk fraction (there were four libraries per milk fraction) were considered as true known miRNAs. With respect to novel miRNA identification, only those with a miRDeep2 score higher than five and at least 10 CPM in two libraries of any milk fraction were retained as true novel miRNAs. The Quantifier module was used to quantify miRNA expression level in each library. The R (v3.0.1) package DESeq2 [33] was used to normalize read counts to account for compositional bias in sequenced libraries and library size and used for miRNA differential expression (DE) analysis.

We further compared the milk miRNome with that of bovine mammary gland tissue. Twelve miRNA sequence datasets of mammary gland tissue that we used for comparison belonged to twelve healthy Canadian Holstein cows fed a total mixed ration of corn and grass silages and concentrates (control diet) from our previous study [17]. The library preparation protocol, sequencing platform (Hiseq 2000) and small RNA sequencing data analysis pipeline were the same as described in this study.

Significantly enriched miRNAs in milk fractions were determined as follows: significantly highly expressed in one milk fraction over the other two fractions (log2 fold change > 2, FDR < 0.05) and with an average expression level of at least 200 CPM in the enriched milk fraction. Significantly enriched miRNAs in mammary gland tissue were determined to be those with a significantly higher expression in mammary gland tissue than in all the three milk fractions (log2 fold change > 2, FDR < 0.05) and with an average expression level of at least 200 CPM in mammary gland tissue.

The target genes of top expressed miRNAs as well as enriched miRNAs in milk fractions were predicted using Ingenuity Pathway Analysis (IPA) software (Ingenuity Systems Inc., USA). MiRNA target prediction information in IPA database is very comprehensive as it includes not only bioinformatics predictions using TargetScan (www.targetscan.org), but also from experimentally validated information on gene-miRNA interactions from TarBase database (http://www.microrna.gr/tarbase) and miRecords (http://mirecords.biolead.org/). The miRNA target filter function of IPA enabled us to focus on the target genes that were experimentally observed or predicted with high confidence. Predicted gene targets of miRNAs were then subjected to function and pathway analysis using IPA core analysis function.

### Real time quantitative PCR (qPCR)

Total RNA from the same sample used in miRNA-sequencing was reverse transcribed using Universal cDNA Synthesis Kit II from Exiqon (Exiqon Inc., USA). The cDNA was then diluted in 9 volumes of nuclease-free water and subjected to quantitative qPCR on a Stepone Plus System (Applied Biosystems, USA) using an ExiLENT SYBR^®^ Green Master Mix Kit (Exiqon, USA) and the miRCURY LNA^™^ Assay (Exiqon, USA) according to the manufacturer’s instructions. The comparative Ct (ΔΔCt) method was used to determine the expression level of miRNA. The geometric mean of bta-miR-103 and bta-miR-25 was used as endogenous control.

## Results

### Total RNA extraction

The total RNA concentration in milk fat was 173.4 ± 50.1 ng/mL milk (mean ± SD) which was similar with that of milk cells (183.0 ± 58.4 ng/mL milk) while the RNA concentration of milk whey was much lower (86.3 ± 42.3 ng/mL milk). Our extraction protocol achieved a good purity of extracted RNA with 260/280 ratios ranging from 2.03 ± 0.11 in whey to 2.12 ± 0.03 in fat and cells (Table 1).

**Table 1:** Concentration and purity of total RNA extracted from milk fat, whey and somatic cells.

	Cow number	Concentration (ng/ml)	OD260/280	OD260/230	RNA integrity number
**Milk fat**	**12**	242.3	2.1	2.1	2.2
	**16**	173.5	2.1	2.1	2.5
	**710**	125	2.2	2.3	NA
	**7134**	152.8	2.1	2.1	3.2
**Milk whey**	**12**	147.5	2	1.6	2.7
	**16**	73.1	2	0.7	2.6
	**710**	49.9	1.9	1.1	NA
	**7134**	74.8	1.9	1.3	2.7
**Milk cell**	**12**	145.2	2.1	1.8	7.3
	**16**	136	2.2	1.9	8.3
	**710**	264.2	2.1	1.9	8
	**7134**	186.6	2.1	2	6.7

Note: NA = Not available (not determined by Bioanalyzer 2100).

**Fig 1:**
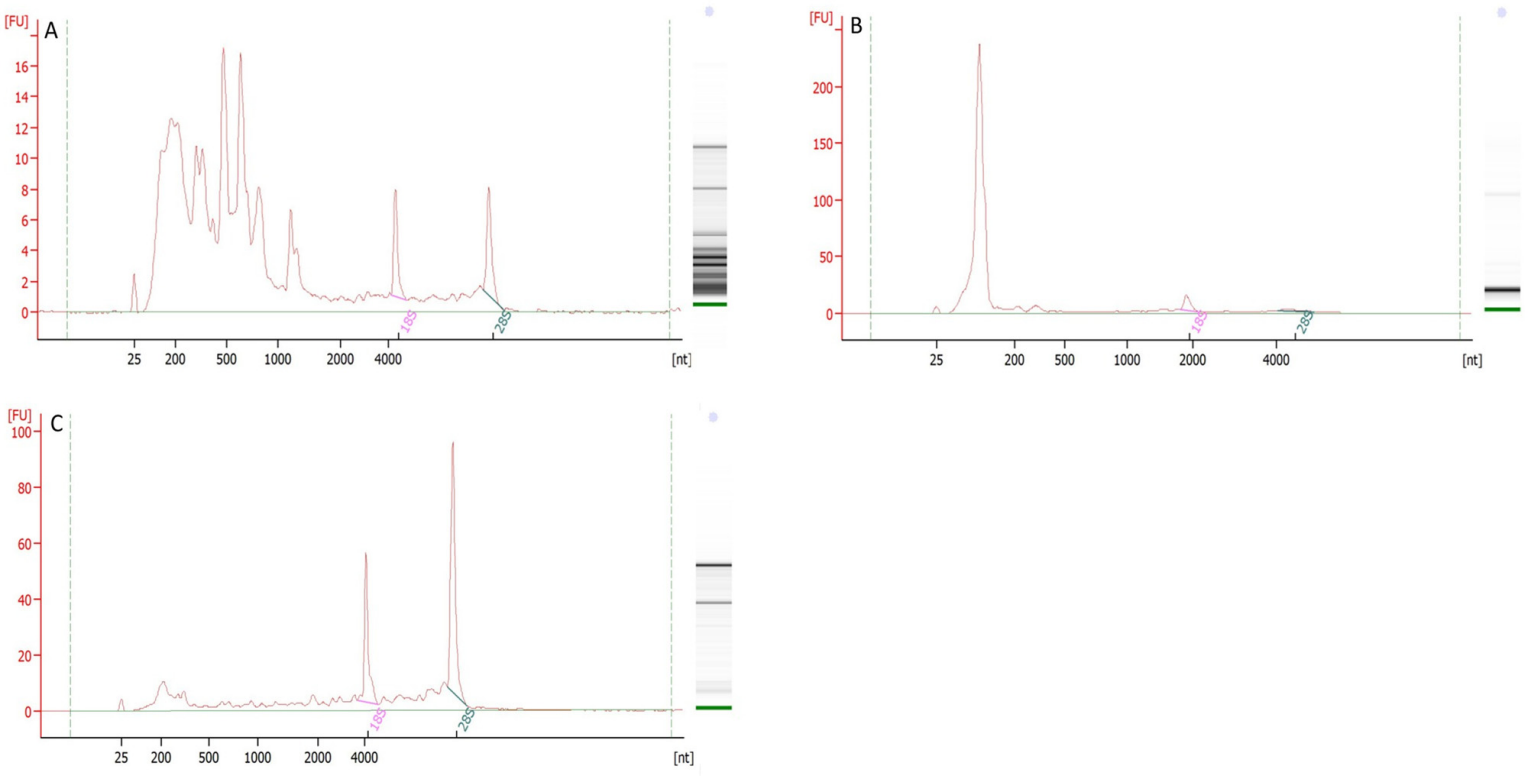
Total RNA capillary electrophoresis electropherograms from milk fractions on Bioanalyzer 2100 (cow No.12). (A) milk fat; (B) milk whey; and (C) milk cells.

We further investigated the RNA integrity (RIN) of the total RNA from the different milk fractions (Table 1, Fig 1). The RIN value of total RNA from milk fat was 2.63 ± 0.51, containing a large amount of low molecular weight fragments apart from the ribosomal RNA fragments which undermined the RIN value. Total RNA from milk whey demonstrated a RIN value of 2.67 ± 0.06, very low amounts of 18S and 28S rRNA on the electropherogram and a sharp peak between 25 nt and 200 nt (Fig 1). Milk cells yielded the highest RIN value of 7.58 ± 0.72.

### MiRNA expression in milk fractions

**Fig 2:**
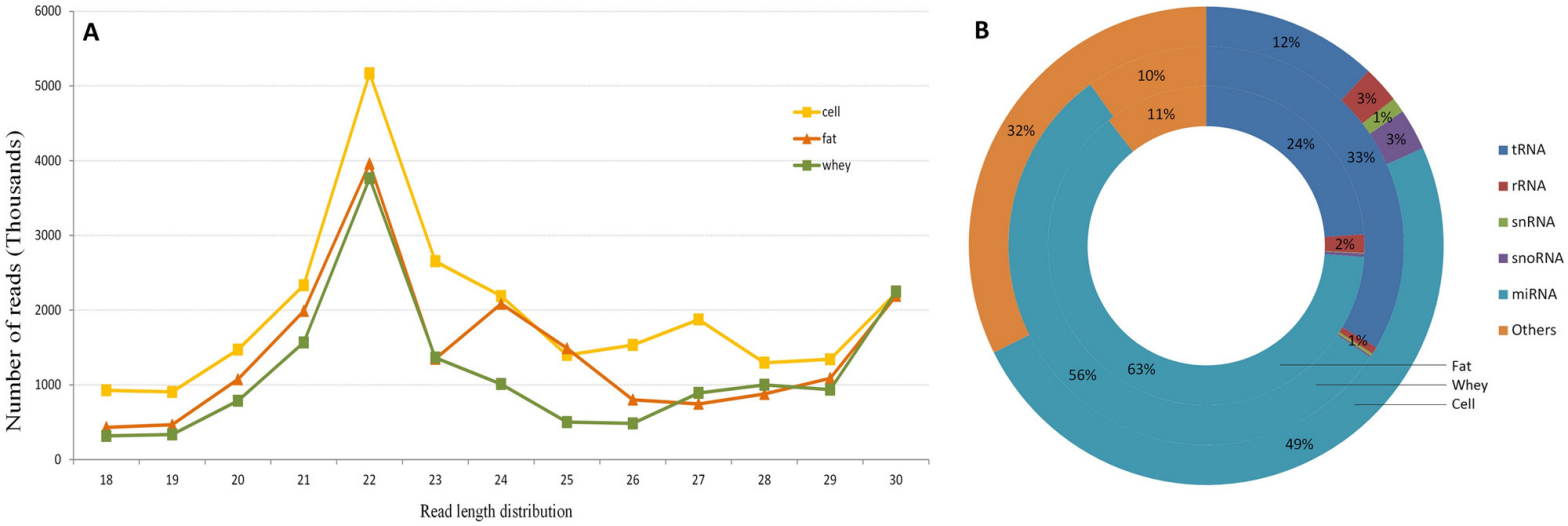
Small RNA length distribution (A) and small RNA species (B). RNA species with a proportion lower than 1% were not labeled in B.

The twelve libraries yielded a total of 164.5 million reads of which 159.5 million clean reads were retained with high quality (S1 Table). Sixty-one million reads with length ranging from 18 to 30 nt were retained for miRNA analysis of which 38.7 million reads were uniquely mapped to the bovine genome. Read length distribution showed that majority of the mapped reads was around 22 nt (Fig 2A). The proportion of reads belonging to other small RNA categories (rRNA, tRNA, snRNA and snoRNA) were respectively 36.1%, 34.5% and 18.3% in fat, whey and cells (Fig 2B). tRNAs were in the majority in all the fractions (fat, whey and cells). Furthermore, the proportion of each small RNA species varied among the three fractions with whey containing the largest ratio of tRNA and cells containing the largest percentage of unclassified small RNA, rRNA, snRNA and snoRNA.

**Fig 3:**
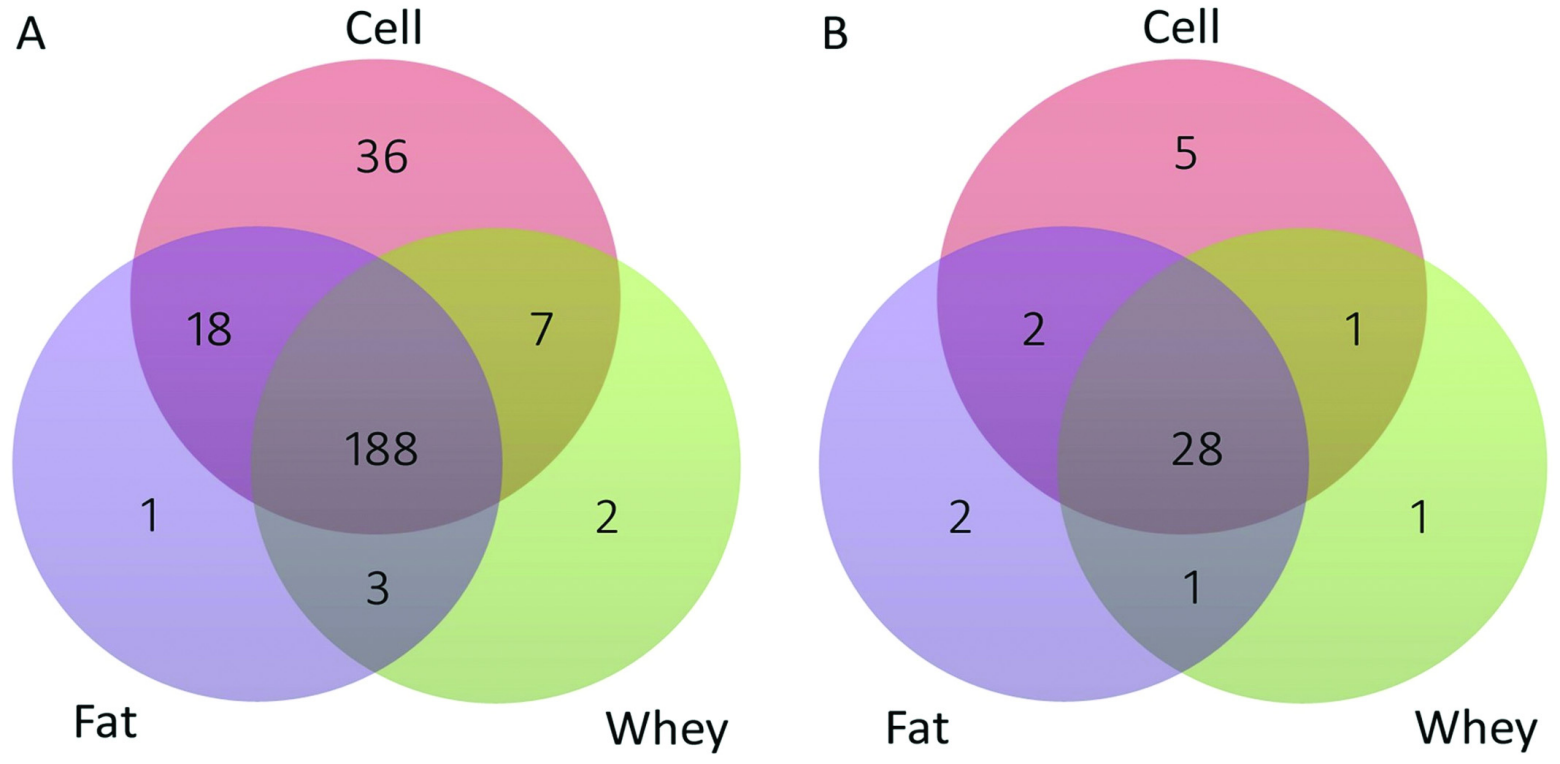
Venn diagrams showing the number of known (A) and novel miRNAs (B) identified in milk fractions as well as miRNAs unique to each fraction or common to two or all three fractions.

We identified 210, 200 and 249 known miRNAs in milk fat, whey and cells, respectively (S2 Table). Most of the miRNAs (188) were shared among all three fractions, while 36 miRNAs were specific to cells and only one and two miRNAs were specific to fat and whey respectively (Fig 3A). MiRdeep2 score of 5 was chosen for novel miRNA prediction, as it yielded a novel miRNA true positive rate of 87±4% to 89±4% and a false positive rate of 6±2 to 11±4 as well as an estimated signal-to noise ratio of 18.2 to 22.8 in the three milk fractions (S3 Table). Based on our stringent criteria, we identified 33, 31 and 36 novel miRNAs in milk fat, whey and cells, respectively (Fig 3B, S4 and S5 Tables). Twenty-eight novel miRNAs were shared by the three fractions while five were unique to cells, two to fat and one to whey.

### Correlations of the milk miRNomes

**Fig 4:**
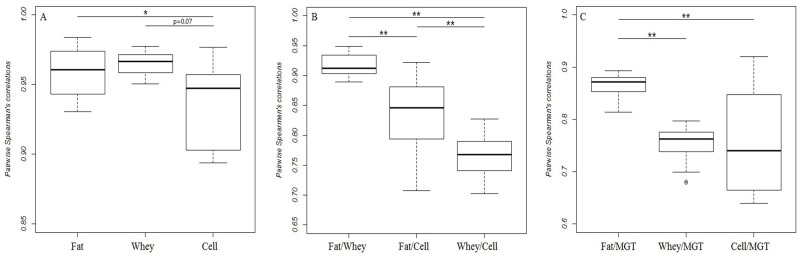
MiRNome homogeneity within milk fractions (A), miRNome similarity among milk fractions (B) and miRNome similarity of milk fractions with mammary gland tissue (C) using Spearman’s correlations. Fat: milk fat; Cell: milk somatic cells; MGT: mammary gland tissue.

Transcriptome from different milk fractions might differ in homogeneity due to the differences and number of the cell types in the milk fractions. Thus we examined the miRNome similarity of every possible pair of samples within each milk fraction using a Spearman’s correlation (Fig 4A). The results showed that samples within each milk fraction were highly correlated with each other. MiRNomes from milk whey were most highly correlated with each other (r_mean_ = 0.965) and with highest consistency followed by milk fat with a slightly lower correlation (mean = 0.959). In contrast, the correlation of the transcriptomes from milk cells was lower (mean = 0.938) than those derived from milk fat (p < 0.05) or milk whey (p = 0.07). It was also evident that transcriptomes of milk whey samples showed high consistency whereas those of milk cells showed a higher heterogeneity.

**Fig 5:**
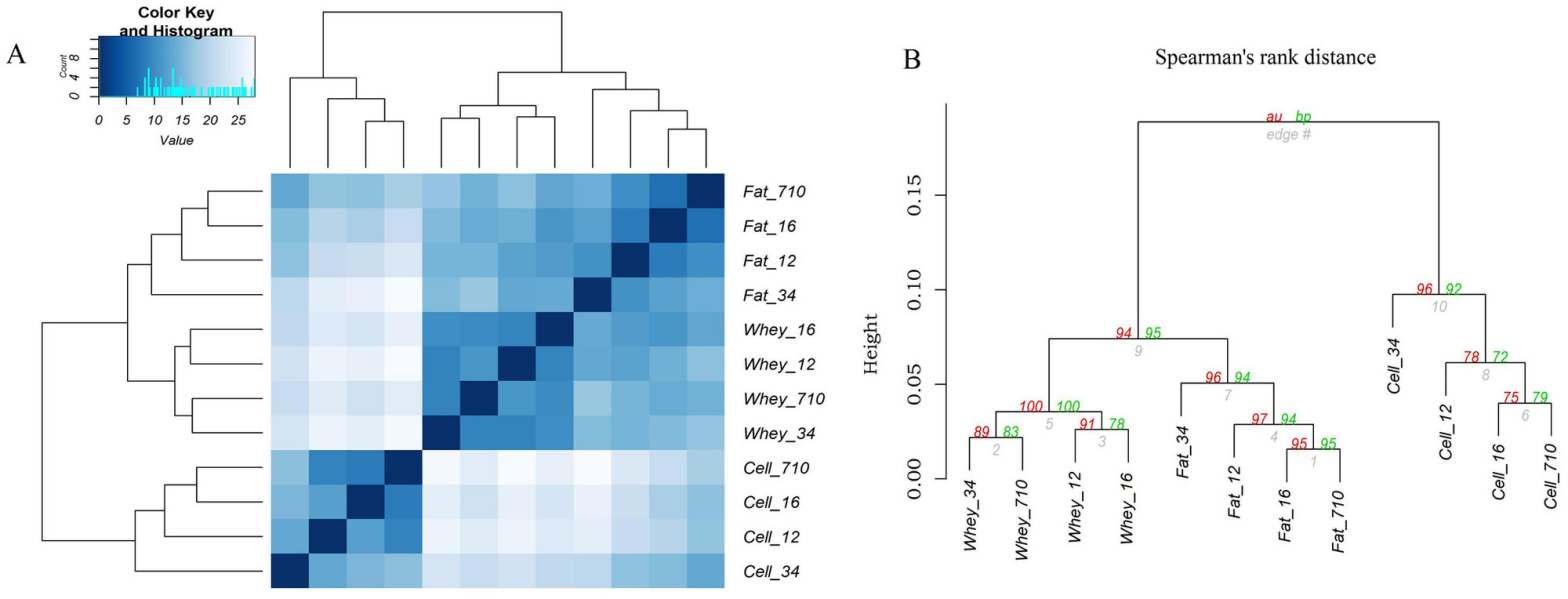
Clustering of milk samples using Hierarchical clustering (Spearman’s rank correlation) (6A) and Pvclust with bootstrap analysis (1000 times) (6B). Normal bootstrap resampling values are represented in the Fig by green letters "bp", for "bootstrap probability". The multi-scale bootstrap resampling probabilities are represented by red letters "au", for "approximately unbiased", and are generally preferred over the "bp" bootstrap probabilities.

We next examined the similarities among the three milk fractions. The correlation between milk fat and whey (fat/whey = 0.917) was significantly higher (p < 0.05) than the other two pairs (fat/cell = 0.835; whey/cell = 0.765) (Fig 4B). Whey/cell demonstrated the lowest correlation (mean = 0.765) compared with the other two pairs (fat/whey, fat/cell). The similarities between the three milk fractions were further analyzed using hierarchical cluster analysis. Results indicated a clear separation of the three fractions into three distinct clusters (Fig 5A). Fat and whey samples were more closely clustered than cell samples, which clustered distinctly from the other two fractions. Bootstrap analysis (1000 times) using Pvclust showed that our hierarchical cluster analysis was with high reliability (Fig 5B).

### Similarity of the milk fraction miRNomes with those of mammary gland tissues

**Fig 6:**
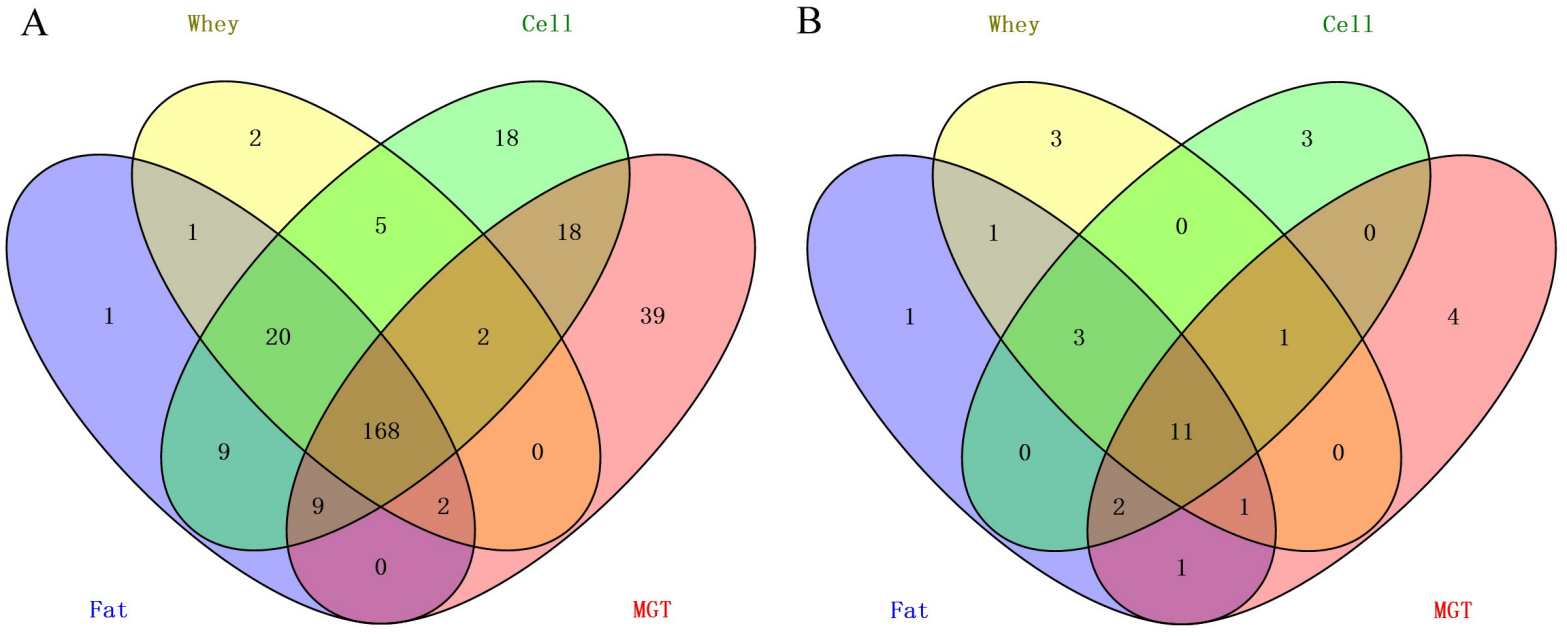
Overlapping of all detected known miRNAs (A) and top 20 expressed miRNAs in milk fractions (fat, whey and cells) and mammary gland tissue (MGT) (B).

**Fig 7:**
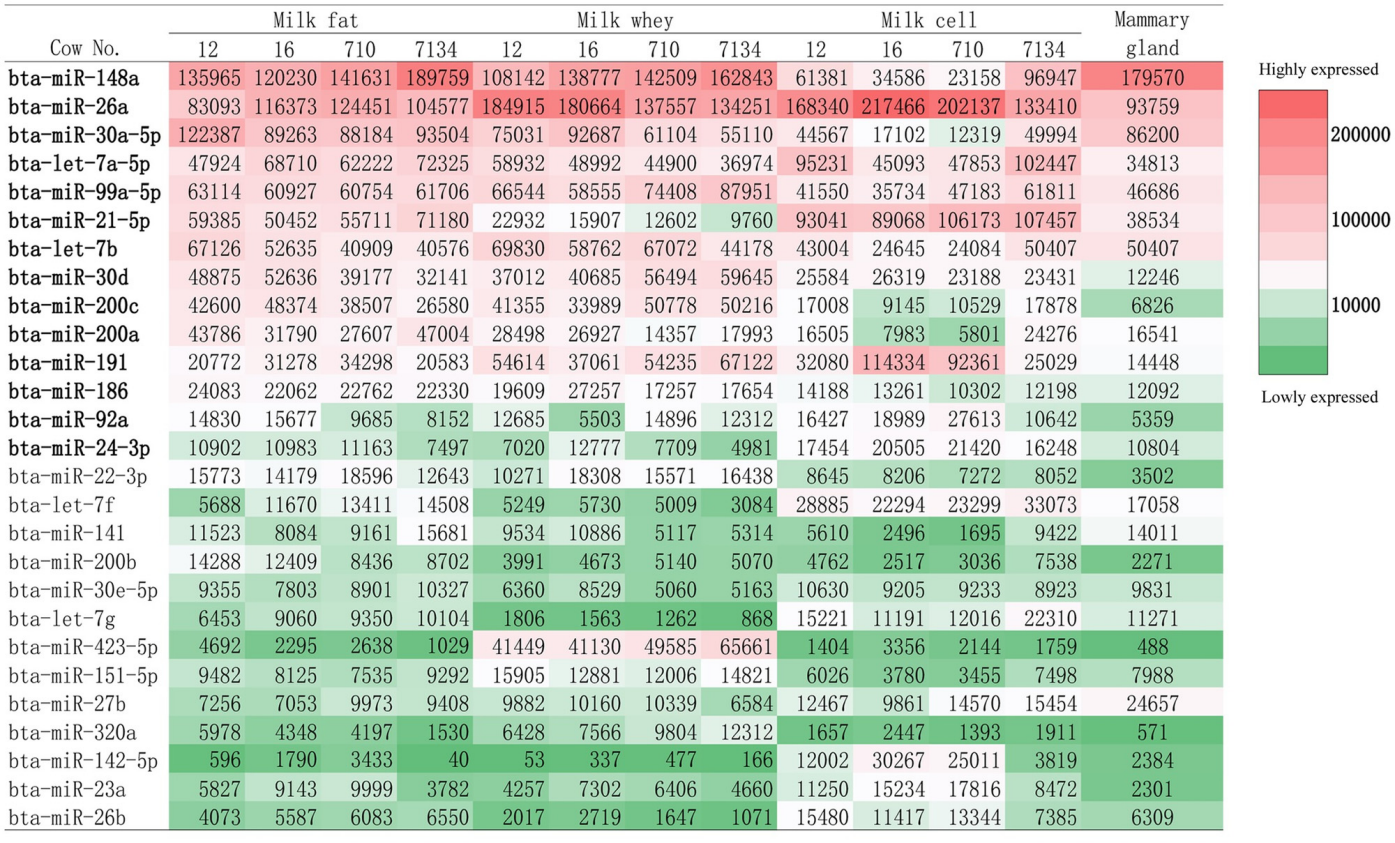
Most highly expressed miRNAs in cow milk fat, whey, cells and mammary gland tissues expressed in count per million (CPM). Red color represents the most highly expressed miRNAs while green color represents relatively lowly expressed miRNAs with lighter color intensities as intermediate highly expressed miRNAs. Underlined CPM numbers indicate that the corresponding miRNAs are among the top 20 expressed miRNAs in the corresponding milk fraction or mammary gland tissue. Four miRNAs that were only among the top 20 expressed miRNAs in mammary gland tissue are not shown.

When the milk fraction miRNomes were compared with that of mammary gland tissue, 168 miRNAs were shared among the four parts comprising 80%, 84%, 97.5% and 52.3% of the fat, whey, cells and mammary gland tissue miRNomes (Fig 6A). Furthermore, 20 miRNAs were shared by the three milk fractions, 18 were shared by milk cells and mammary gland tissue while 18 and 39 were unique to milk cells and mammary gland tissue, respectively. The top 20 expressed miRNAs in each milk fraction (milk fat, whey, cells) and mammary gland tissue accounted for 84.4%, 87.5%, 78.5% and 82.3% of the total read counts respectively, implying essential regulatory roles of these top expressed miRNAs. Further comparison showed that 11 top expressed miRNAs (bta-miR-148a, miR-26a, miR-30a-5p, let-7a-5p, miR-99a-5p, miR-21-5p, miR-30d, miR-200a, miR-191, miR-186, miR-24-3p) were shared among the three milk fractions and mammary gland tissue, three (bta-let-7b, miR-92a, miR-200c) were shared by the three milk fractions while 3 (bta-miR-423-5p, miR-151-5p and miR-320a), 3 (bta-miR-142-5p, miR-23a and miR-26b) and 4 miRNAs (bta-miR-125b, miR-145, miR-10b, miR-143) were unique to milk whey, cells and mammary gland tissue, respectively (Figs 6B and 7).

We then explored which milk fraction miRNome profile was closer to that of mammary gland tissue using Spearman’s correlation (Fig 4C). MiRNome of milk fat showed the highest similarity with mammary gland tissue (r_mean_ = 0.866) while milk whey and cells showed a lower similarity (r_mean_ = 0.755, 0.757 respectively, p < 0.01) with mammary gland tissue. The highest heterogeneity was between milk cells and mammary gland tissue.

### Function of highly expressed milk miRNAs in milk fractions

The fourteen miRNAs that were highly expressed in the three milk fractions (Fig 7) were imported into IPA software to explore their potential biological functions. The fourteen miRNAs were predicted to target 1802 mRNAs with high confidence using IPA target filter. The target genes were enriched in 22 molecular functions including cellular related functions (cellular growth and proliferation, cell death and survival, cellular movement, cellular development, etc.), protein synthesis and metabolism of carbohydrate and lipid (Table 2). When probing into functions related to physiological system development (Table 3), 18 out of the 25 identified functions were associated with organ/tissue/embryonic development or the development and function of hematological system, cardiovascular system, skeletal and muscular system, etc. Functions related with immune cell trafficking and cell-mediated immune response were also enriched. The enriched functions in the category of disease agreed with the category of physiological system development, showing that most diseases (17/22) were associated with developmental disorders (Table 4). Top canonical pathways of the 14 miRNAs included axonal guidance signaling, protein kinase A signaling, cardiac hypertrophy signaling, glucocorticoid receptor signaling and breast cancer regulation by stathmin1 (S6 Table).

**Table 2:** Significantly enriched molecular functions by the target genes (1802) of 14 highly expressed miRNAs in milk fractions.

Category	p-value	#Molecules
Cellular growth and proliferation	1.26E-195–6.91E-29	1004
Cell death and survival	1.3E-170–3.17E-28	907
Cellular Movement	1.1E-145–2.08E-29	678
Cellular Development	2.95E-136–2.27E-28	917
Cell Morphology	5.63E-119–2.07E-28	685
Gene expression	2.87E-114–3.73E-41	689
Cellular function and maintenance	2.74E-101–2.07E-28	700
Cell cycle	4.14E-84–2.97E-29	424
Cellular assembly and organization	2.53E-78–2.07E-28	480
Post-translational modification	2.03E-76–9.19E-29	402
Carbohydrate metabolism	1.84E-57–6.34E-30	356
DNA replication, recombination, and repair	1.08E-53–1.08E-53	162
Lipid metabolism	1.55E-52–1.38E-44	352
Molecular transport	1.55E-52–6.38E-29	567
Small molecule biochemistry	1.55E-52–6.53E-35	470
Cell signaling	1.97E-50–6.38E-29	432
Cell-to-cell signaling and interaction	1.97E-44–1.61E-28	599
Free radical scavenging	5.12E-43–5.29E-30	166
Amino acid metabolism	5.07E-41–5.07E-41	99
Protein synthesis	1.63E-40–1.11E-33	376
Vitamin and mineral metabolism	6.38E-29–6.38E-29	117

**Table 3:** Significantly enriched functions related with physiological system development by target genes (1802) of the 14 highly expressed miRNAs in milk fractions.

Category	p-value	#Molecules
Organismal development	6.33E-104–3.56E-28	949
Tissue development	5.69E-63–3.56E-28	841
Organismal survival	1.15E-181–9.25E-41	803
Embryonic development	6.33E-104–3.56E-28	690
Tissue morphology	1.3E-108–1.37E-28	680
Organ development	5.69E-63–3.56E-28	620
Hematological system development and function	3.4E-63–2.01E-30	513
Cardiovascular system development and function	1.11E-122–1.36E-29	479
Skeletal and muscular system development and function	5.69E-63–1.62E-29	464
Connective tissue development and function	2.27E-60–6.97E-30	451
Behavior	3.24E-86–8.65E-30	375
Organ morphology	1.07E-56–1.36E-29	365
Reproductive system development and function	4.31E-56–9.73E-38	313
Digestive system development and function	1.55E-50–1.98E-33	300
Lymphoid tissue structure and development	4.38E-60–3.56E-28	293
Hematopoiesis	4.38E-60–2.01E-30	287
Immune cell trafficking	1.06E-50–3.42E-38	251
Hair and skin development and function	2.88E-43–2.27E-28	246
Nervous system development and function	6.05E-45–1.37E-39	199
Respiratory system development and function	1.17E-42–5.51E-35	169
Visual system development and function	3.57E-36–3.57E-36	148
Endocrine system development and function	5.25E-37–5.25E-37	137
Cell-mediated immune response	2.02E-45–9.18E-45	133
Renal and urological system development and function	2.66E-30–3.22E-29	102
Hepatic system development and function	3.38E-39–1.07E-38	90

**Table 4:** Significantly related diseases of the gene targets (1802) of the 14 highly expressed miRNAs in milk fractions.

Category	p-value	#Molecules
Cancer	2.43E-84–3.79E-28	1668
Organismal injury and abnormalities	3.4E-73–3.79E-28	1677
Infectious diseases	7.99E-73–9.37E-29	374
Developmental disorder	1.5E-64–6.51E-33	590
Reproductive system disease	1.18E-62–4.22E-29	988
Skeletal and muscular disorders	8.46E-62–5.84E-30	694
Hematological disease	5.34E-59–1.39E-28	529
Tumor morphology	7.43E-58–2.18E-33	279
Metabolic disease	1.16E-55–3.55E-31	426
Inflammatory response	6.6E-53–1.03E-35	422
Gastrointestinal disease	1.74E-52–3.79E-28	1329
Neurological disease	6.11E-52–5.84E-30	564
Cardiovascular disease	3.54E-50–9.33E-31	287
Connective tissue disorders	4.49E-50–5.34E-42	402
Inflammatory disease	3.9E-44–7.21E-39	335
Endocrine system disorders	4.2E-43–5.67E-30	430
Immunological disease	1.7E-40–1.39E-28	482
Nutritional disease	3.21E-37–3.21E-37	150
Psychological disorders	6.16E-36–7.12E-29	354
Hepatic system disease	6.04E-34–3.79E-28	788
Respiratory disease	6.25E-34–5.25E-29	270
Hereditary disorder	6.89E-33–5.84E-30	287

### Milk and mammary gland tissue enriched miRNAs

Apart from the high similarity between milk fractions, we further explored the relationship between highly expressed miRNAs in each milk fraction and mammary gland tissue. Our analysis revealed that three miRNAs were highly enriched (high expression level, log2 fold change > 2, FDR < 0.05) in whey and 11 in milk cells (Table 5). In contrast, no miRNA were specifically enriched in milk fat. Another 14 miRNAs demonstrated a high enrichment in mammary gland tissues and low/no expression in the three milk fractions. Interestingly, bta-miR-221/miR-222, and bta-miR-143/145 which were abundantly expressed in milk cells are located close to one another, within one chromosomal cluster on chromosome X. Furthermore, bta-miR-199a-5p and miR-199b which are from the same miRNA family are enriched in mammary gland tissue. These results suggest that the specifically enriched miRNAs might be co-expressed and co-secreted. Some of the specifically enriched miRNAs were further verified using real-time quantitative PCR.

**Table 5:** Milk fractions and mammary gland tissue (MGT) highly enriched miRNAs (in bold face and underlined), expressed in CPM.

		Milk fat				Milk whey				Milk cell				MGT
	Cow No.	12	16	710	7134	12	16	710	7134	12	16	710	7134	
Milk whey	bta-miR-423-5p	4691	2295	2637	1029	**41438**	**41117**	**49571**	**65649**	1404	3356	2143	1759	488
	bta-miR-2478	63	110	80	52	**490**	**166**	**1850**	**904**	94	325	219	100	172
	bta-miR-EIA10_2262	239	78	81	180	**1955**	**1866**	**1830**	**1008**	76	176	77	231	0
Milk cells	bta-miR-142-5p	596	1789	3431	40	53	337	476	166	**12001**	**30265**	**25009**	**3819**	2384
	bta-miR-10a	34	32	45	5	7	27	29	12	**329**	**649**	**645**	**198**	3359
	bta-miR-146a	294	994	1790	36	23	96	63	26	**5346**	**6086**	**5514**	**1518**	1231
	bta-miR-205	58	39	48	6	5	23	49	15	**254**	**264**	**397**	**330**	1463
	bta-miR-223	125	341	653	3	9	21	16	10	**6533**	**9801**	**11038**	**655**	9
	bta-miR-221	187	520	805	9	23	218	162	35	**2175**	**2778**	**3240**	**977**	256
	bta-miR-150	22	28	160	11	15	10	49	25	**3307**	**3888**	**4875**	**2143**	145
	bta-miR-222	139	359	622	12	11	62	130	32	**2067**	**4580**	**3604**	**778**	176
	bta-miR-155	54	114	198	31	130	109	149	101	**1211**	**1811**	**1694**	**404**	109
	bta-miR-224	20	14	54	4	9	13	36	10	**226**	**170**	**245**	**319**	257
	bta-miR-31	91	31	42	10	3	13	5	1	**206**	**171**	**241**	**676**	1
MGT	bta-miR-143	243	246	217	89	90	127	114	106	482	275	238	579	**118288**
	bta-miR-10b	23	190	132	76	41	185	203	85	7	33	19	38	**59944**
	bta-miR-145	58	63	68	14	14	51	43	37	126	127	72	100	**11993**
	bta-miR-100	19	45	51	16	22	36	61	44	65	123	179	136	**9156**
	bta-miR-199a-5p	0	0	0	0	2	3	5	1	1	1	4	0	**7407**
	bta-miR-126-3p	3	2	3	4	3	1	4	2	3	3	3	9	**7004**
	bta-miR-126-5p	0	0	0	0	0	0	0	0	0	0	0	0	**5161**
	bta-miR-199b	0	0	0	0	2	0	0	1	23	1	10	0	**3790**
	bta-miR-127	182	192	23	42	448	237	42	114	66	46	6	20	**3780**
	bta-miR-96	1558	1379	1470	2725	1278	1629	1022	685	1245	692	773	2228	**3549**
	bta-miR-199a-3p	4	3	2	2	0	2	2	1	67	1	17	2	**3425**
	bta-miR-1468	124	191	173	109	261	217	389	388	179	213	209	93	**1623**
	bta-miR-101	303	277	271	509	132	119	66	41	416	345	344	516	**1427**
	bta-miR-195	0	0	0	0	1	1	1	1	8	9	11	28	**635**

The whey, cell and mammary gland enriched miRNAs were predicted to target 1894, 3314 and 5775 genes respectively using IPA miRNA target filter. These targets genes were further subjected to the IPA core analysis to explore their potential related biological functions. The majority of the significantly enriched molecular functions, physiological and development functions, and diseases were shared among the milk fractions and mammary gland tissue (S2–S4 Figs, S7 Table). Most exclusive functions were found in whey (cellular compromise, drug metabolism, lipid metabolism, immune cell trafficking, organismal functions, auditory and vestibular system development and function, dermatological diseases and conditions, inflammatory disease, metabolic disease). In contrast, cell fraction had two exclusively enriched functions (humoral immune response and nutritional disease) while mammary gland tissue had none except for functions shared with milk fraction.

### Validation of miRNA expression by qPCR

**Fig 8:**
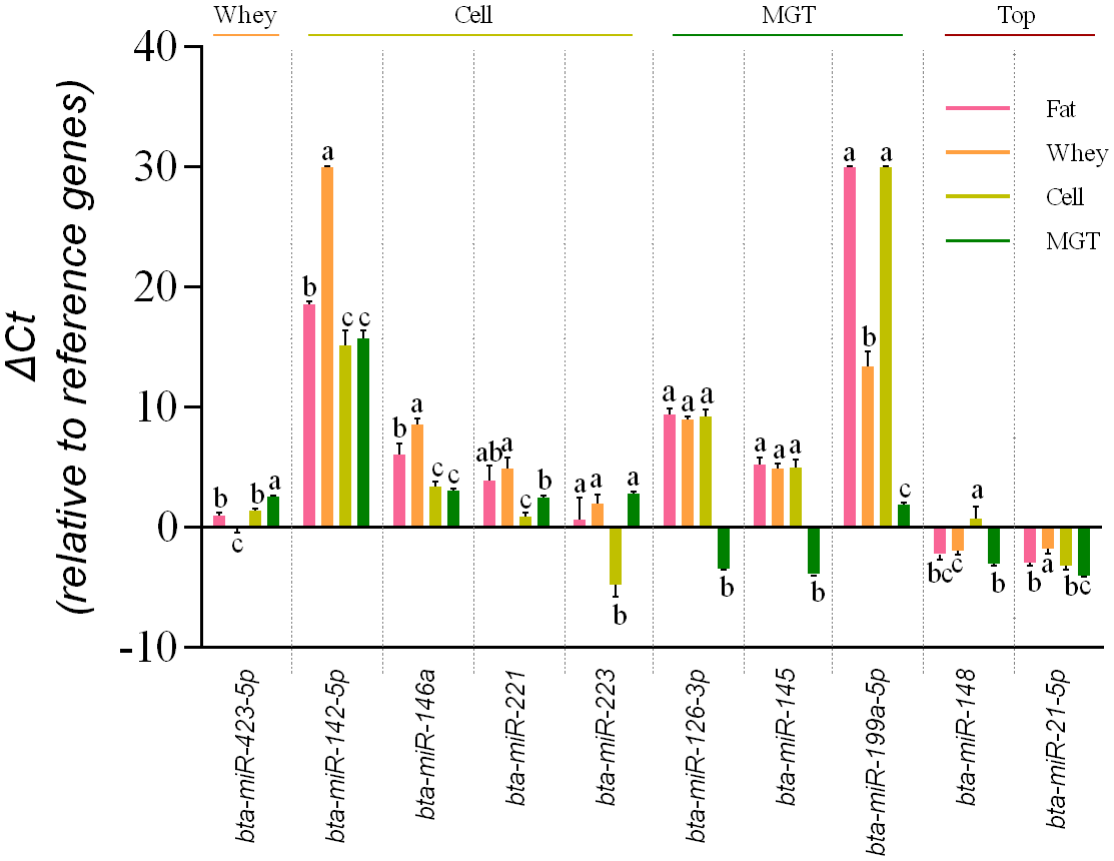
qPCR validation of two top expressed miRNAs as well as milk whey (one), cell (four) and mammary gland tissue (MGT) (three) enriched miRNAs.

Real time qPCR was used to validate the expression of two top expressed miRNAs as well as eight highly enriched miRNAs in milk fractions and mammary gland tissue (Fig 8). qPCR results of the two top expressed miRNAs (bta-miR-148, miR-21-5p) were consistent with results of miRNA-sequencing, demonstrating the highest level of expression in the corresponding milk fraction and in mammary gland tissue. Bta-miR-423-5p which was found to be a whey enriched miRNA by DE analysis showed the highest expression in whey (p < 0.05) while the four cell enriched miRNAs (bta-miR-142-5p, miR-146a, miR-221, miR-223) were most highly expressed in milk cells than in the other two milk fractions (p < 0.05). Statistically, bta-miR-142-5p and miR-146a demonstrated a comparable expression level between milk cell and mammary gland tissue. In addition, the expression of three mammary gland tissue enriched miRNAs (bta-miR126-3p, miR-145-5p and miR-199a-5p) was significantly higher in mammary gland tissue than in the three milk fractions (p < 0.05).

## Discussion

In this study, we have successfully isolated total RNA from different milk fractions including fat, whey and cells for small RNA-Seq library preparation. Our data showed differences/similarities between the miRNomes of the milk fractions. The differences/similarities mainly stem from the milk secretion process which decides the RNA origin of each fraction. Milk fat globules can trap cytoplasmic crescents of mammary epithelial cells during fat secretion by mammary epithelial cells [14], which explains the high correlation between the miRNome of milk fat and mammary gland tissue in this study. Milk somatic cells are more heterogeneous consisting of both immune and exfoliated epithelial cells which are shed into milk from the udder epithelium. In milk whey, there are exosomes which are released into milk by epithelial cells [2]. The epithelial cells which are shed into milk are usually dead cells and thus cannot fully depict the true metabolic state of MECs [34]. Instead, milk fat and whey contains RNA from secreting MECs and might be a better source of mammary RNA [25].

Total RNA concentrations were higher in milk fat and somatic cells than in the whey fraction as has been found in a previous study [12]. The RNA integrity of somatic cells was the highest while those of milk fat and whey were much lower. The low RNA integrity in milk fat could be due to its vulnerability to degradation [35] but is more likely a result of contaminant from bacteria sequences [36] (RNA or low molecular weight fragments between 5S region and 18S region on electrophoretic electropherogram, Fig 1). However, the small RNAs from bacteria range in length from 50 to 500 nt [37] which is out of the detection range of the small RNA library preparation and sequencing method used. Nevertheless, when small RNAs were separated from the total RNA, we could see a typical peak corresponding to the small RNA fraction, an indication that this fraction was intact (S1 Fig), as have been observed in our previous RNA extraction from tissue samples (data not shown). The low RNA integrity of milk whey, with a sharp peak between 25nt and 200nt in the electropherogram is due to abundant amounts of low weight molecules including 5S rRNA and small RNAs as compared to much lower amounts of 18S and 28S rRNA (Fig 1C). This observation is supported by previous findings [2,7,11,38] where a dominant small RNA proportion was found in milk whey.

The miRNA profile of bovine milk fat and whey as well as mammary gland tissue/mammary epithelial cells has been determined using high throughput sequencing in several studies with various number of miRNAs (novel and known) detected. For instance, Chen et al. [23] identified 245 miRNAs from milk plasma (whey); Jin et al. [39] reported 245 miRNAs in MAC-T cells (a bovine mammary epithelial cell line) while Bu et al. [40] reported 388 known miRNAs in a bovine mammary epithelial cell line of Chinese Holstein cattle origin. In our recent study [17], 321 known miRNAs were identified in bovine mammary gland tissue. Although the number of identified miRNAs differed in these studies, many of the top expressed miRNAs in this study are also amongst top highly expressed miRNAs in previous studies on bovine milk either from whey [4], mammary epithelial cells [39], whey exosomes [16] or mammary gland tissue [17,41,42] (Table 6). These highly, commonly expressed miRNAs observed in various studies suggest that they may have critical regulatory roles in bovine mammary gland development/productivity and possibly, to milk recipients (humans).

**Table 6:** Highly expressed miRNAs in this study are also among abundantly expressed miRNAs in previous studies on bovine milk and mammary gland tissue/epithelial cells.

	Chen et al (2010)^[^^4^^]^	Jin et al (2014)^[^^39^^]^	Li et al (2012)^[^^43^^]^	Le Guilou et al (2014)^[^^41^^]^	Li et al (2015)^[^^42^^]^	Sun et al (2015)^[^^16^^]^
Tissue type	milk whey	MAC-T^1^	MGT^2^	MGT	MGT	Milk exosome
Total miRNA number	245	231	298	167	321	242
bta-miR-148a	√^3^	√	√	√	√	√
bta-miR-26a	×^4^	√	√	√	√	√
bta-miR-30a-5p	√	×	√	√	√	√
bta-let-7a-5p	√	√	√	√	√	√
bta-miR-99a-5p	√	×	√	√	√	×
bta-miR-21-5p	√	√	√	√	√	√
bta-let-7b	√	×	√	√	√	√^5^
bta-miR-30d	√	√	×	√	√	√
bta-miR-200c	√	√	√	√	√	√
bta-miR-200a	×	×	√	√	√	√
bta-miR-191	×	√	√	√	√	√
bta-miR-186	×	√	√	√	√	√
bta-miR-92a	√	√	√	√	√	√
bta-miR-24-3p	√	×	×	√	√	×

^1^MAC-T: a bovine mammary epithelial cell line,

^2^MGT: mammary gland tissue,

^3^√ Indicate highly expressed miRNAs in those studies (Top 30).

^4^× Indicate lowly expressed miRNAs in those studies (>Top50);

^5^√ indicate medium expressed miRNAs (Top30-50) in those studies.

One of our primary aims was to determine whether milk sampling could be a non-invasive replacement of biopsy sampling to study the miRNome of mammary gland tissue. The biopsy approach is the method of choice to harvest mammary gland tissues for monitoring the miRNome activities of mammary epithelial cells *in vivo* but it is invasive, expensive, technically challenging and could predispose animals to disease pathogens. A non-invasive method is thus much needed to enable easy collection of samples and repeat analysis with minimal damage to cow health. mRNA isolated from milk fat and somatic cells generally demonstrated a similar mRNA transcriptome with mammary gland tissue and appear to be the simplest way to assess the transcriptomes of mammary gland [25,35]. We thus explored whether it would hold for miRNA profiles. A distinct separation of miRNA expression among the three milk fractions was observed using the hierarchical cluster analysis. All samples clustered together by milk fractions instead of by cows, with fat and whey samples being closer. The majority of the detected known and novel miRNAs were shared among the three milk fractions with 14 out of 20 top expressed miRNAs common to all three fractions. Also, milk fat miRNome showed the highest similarity with that of mammary gland tissue followed by whey. The lowest similarity was observed between milk cells and mammary gland tissue which agrees with the heterogeneity of somatic cells. The fat fraction appeared to be enriched with RNA mostly from mammary epithelial cells [35]. Therefore, we proposed that milk fat could be the best alternative to mammary gland tissue in assessing the transcriptomic activities of mammary epithelial cells at the miRNA level. However, it should also be noted that the mammary gland tissue is complex and heterogeneous with multiple cell types including mammary epithelial cells, myoepithelial, stromal and immune cells [44] which explains the degree of observed correlation with milk fat miRNome as compared to the other fractions and could potentially cause the miRNome difference of mammary gland with milk.

MiRNAs in milk, especially in fat and whey fractions are secreted by mammary epithelial cells and thus support their involvement in mammary gland development and milk synthesis. Increasing evidence has highlighted the essential roles of miRNAs in regulating mammary gland development and milk synthesis [17,39,45,46]. As expected, functional analysis of the 14 commonly and highly expressed miRNAs in the milk fractions showed that many of the molecular functions were related to milk synthesis, e.g. fatty acid metabolism, protein synthesis, amino acid metabolism, in addition to housekeeping related functions like cellular functions (Table 2). The IPA function analysis tools not only provided us the enriched molecular functions but also enabled us to dig deeper into the cellular functions associated with physiological system development and disease related functions. We found that the miRNA targets were related with infections like cell-mediated immune response and immune cell trafficking (Table 3), which agrees with the related diseases of the targets of these miRNAs (infectious diseases, inflammatory response and inflammatory disease) (Table 4).

Apart from the similarities in the miRNome profiles of milk and mammary gland tissue, some miRNAs were highly enriched in two milk fractions (3 in whey and 11 in cells) and mammary gland tissue (14). Highly enriched miRNAs might point to important regulatory functions specific to milk fractions or mammary gland tissue. Since highly enriched miRNAs in milk whey are packaged in exosomes, they could potentially exert modulatory functions in infants [47] to other milk recipients. For example, highly enriched miR-423-5p in whey has been previously found to be highly expressed in porcine whey exosomes and could be involved in regulation of the IgA network and immunity of piglets [7]. Interestingly, one whey highly enriched miRNA is a novel miRNA, bta-miR-EIA10_2262, which also belongs to the miR-423-5p family with the same seed region (gaggggc) and thus is expected to target similar genes (with similar functions) as miR-423-5p. Notably, the function enrichment analysis showed that the highest number of exclusively enriched functions were found in whey, suggesting a potential whey miRNA delivery of regulatory mechanisms [38,48], for example through immune cell trafficking, organismal functions, auditory and vestibular system development and function. In mammary gland tissue, highly enriched miR-126-3p regulates lactation and mammary gland development in mouse [49]. However, roles of most of the identified highly enriched miRNAs need to be further elucidated.

Milk is not just food but might represent a sophisticated signaling system that delivers maternal milk-derived messages to promote postnatal health [6,47,50]. Breast milk contains large amounts of miRNAs which is higher than that of other body fluids like plasma [51]. Advances in recent years have shown that cow milk miRNA can be absorbed by humans in meaningful amounts [15] and can affect gene expression in human peripheral blood mononuclear cells [22]. Most enriched physiological functions of gene targets of the top expressed miRNA were related with organ and tissue development (18/25) in the physiological system development category. Besides, they were also implicated in organ developmental disorders within disease category, suggesting that these miRNAs could play important roles in neonatal development. Milk miRNA could also have regulatory roles in many metabolic pathways and in immune development [21]. Even though functions of most of the abundantly expressed miRNAs remain to be verified, roles of several miRNAs have been confirmed in recent years. miR-21-5p mediates suppression of target genes to enhance upstream and downstream mTORC1 (mammalian target of rapamycin complex 1) signaling for postnatal growth [20] while miR-26a can enhance insulin sensitivity and suppress lipogenesis and gluconeogenesis [52]. Two abundantly expressed miRNAs of miR-30 family, miR-30a and miR-30d, have also been considered as regulators in promoting insulin sensitivity [53]. In addition, miR-24-3p is implicated in the development of innate immunity [54] while miR-200 family have roles in promoting antigen-specific T-cell activation [55] and in regulating differentiation and proliferation of neurons [56]. Notably, miR-200c was one of the cow milk miRNAs which was detected in human plasma after meaningful milk consumption [22].

In conclusion, we have presented a comprehensive profile of the miRNome of milk with respect to three fractions-, fat, whey and cells, using high throughput sequencing. Within milk fractions, fat and whey samples clustered closely and demonstrated the highest similarity. Furthermore, the comparison between miRNome from milk fractions with that from mammary gland tissue showed that milk fat would be a good alternative in evaluating the miRNome profile of mammary gland tissue. Our data also showed that highly expressed miRNAs in the three milk fractions could be important regulators of mammary gland function and potentially infant development.

## Supporting Information

S1 Fig Total RNA capillary electrophoresis electropherogram from total RNA as well as the corresponding long/small RNA fractions separated by miRVana kit. (A) Total RNA, (B) long RNA fraction and (C) small RNA fraction from trial sample 1. (D) Total RNA, (E) long RNA fraction and (F) small RNA fraction from trial sample 2. C shows that the small RNA fraction is intact despite evidence of degradation or presence of contaminating bacterial sequences (large peak from 200 to 900 nt in the electropherogram). Furthermore, improved RIN value of long RNA fraction (B and E) depended on the RIN value of the starting material (A and D).
(TIF)

S2 Fig Significantly enriched molecular functions (S2-1), physiological and developmental functions (S2-2) and disease functions (S2-3) of three milk whey specifically enriched miRNAs. (PDF)

S3 Fig Significantly enriched molecular functions (S3-1), physiological and developmental functions (S3-2) and disease functions (S3-3) of 11 milk cells specifically enriched miRNAs. (PDF)

S4 Fig Significantly enriched molecular functions (S4-1), physiological and developmental functions (S4-2) and disease functions (S4-3) of 14 mammary gland tissue specifically enriched miRNAs. (PDF)

S1 Table Read mapping statistcs (S1-1) and read length distribution of the clean reads ranging from 17 nt to 30 nt. (XLSX)

S2 Table Raw read counts of known miRNAs expressed in milk fractions. (XLSX)

S3 Table MiRDeep2 output for fat (S3-1), whey (S3-2) and somatic cell (S3-3) samples. (XLSX)

S4 Table Novel miRNA genomic and sequence information. (XLSX)

S5 Table Raw read counts of novel miRNAs expressed in milk fractions. (XLSX)

S6 Table Most significantly enriched pathways of the target genes of 14 top expressed miRNAs in milk. (XLSX)

S7 Table Significantly enriched biological functions that were specific or common to three milk fractions. (XLSX)

## References

[1] Sun Q, Chen X, Yu J, Zen K, Zhang C-Y, Li L (2013) Immune modulatory function of abundant immune-related microRNAs in microvesicles from bovine colostrum. Protein & Cell 4: 197–210. 23483481 10.1007/s13238-013-2119-9 PMC4875502

[2] Hata T, Murakami K, Nakatani H, Yamamoto Y, Matsuda T, Aoki N (2010) Isolation of bovine milk-derived microvesicles carrying mRNAs and microRNAs. Biochemical and Biophysical Research Communications 396: 528–533. 10.1016/j.bbrc.2010.04.135 20434431

[3] Izumi H, Kosaka N, Shimizu T, Sekine K, Ochiya T, Takase M (2012) Bovine milk contains microRNA and messenger RNA that are stable under degradative conditions. Journal of Dairy Science 95: 4831–4841. 10.3168/jds.2012-5489 22916887

[4] Chen X, Gao C, Li H, Huang L, Sun Q, Dong Y, et al (2010) Identification and characterization of microRNAs in raw milk during different periods of lactation, commercial fluid, and powdered milk products. Cell Research 20: 1128–1137. 10.1038/cr.2010.80 20548333

[5] Gu Y, Li M, Wang T, Liang Y, Zhong Z, Wang X, et al (2012) Lactation-related microRNA expression profiles of porcine breast milk exosomes. PLoS One 7: e43691 10.1371/journal.pone.0043691 22937080 PMC3427246

[6] Zhou Q, Li M, Wang X, Li Q, Wang T, Zhu Q, et al (2012) Immune-related microRNAs are abundant in breast milk exosomes. International Journal of Biological Sciences 8: 118–123. 22211110 10.7150/ijbs.8.118 PMC3248653

[7] Chen T, Xi Q-Y, Ye R-S, Cheng X, Qi Q-E, Wang S-B, et al (2014) Exploration of microRNAs in porcine milk exosomes. BMC Genomics 15: 1–19. 24499489 10.1186/1471-2164-15-100 PMC4008308

[8] Izumi H, Kosaka N, Shimizu T, Sekine K, Ochiya T, Takase M (2014) Time-dependent expression profiles of microRNAs and mRNAs in rat milk whey. PloS One 9: e88843 10.1371/journal.pone.0088843 24533154 PMC3923055

[9] Modepalli V, Kumar A, Hinds LA, Sharp JA, Nicholas KR, Lefevre C (2014) Differential temporal expression of milk miRNA during the lactation cycle of the marsupial tammar wallaby (Macropus eugenii). BMC Genomics 15: 1012 10.1186/1471-2164-15-1012 25417092 PMC4247635

[10] Bartel DP (2009) MicroRNAs: Target Recognition and Regulatory Functions. Cell 136: 215–233. 10.1016/j.cell.2009.01.002 19167326 PMC3794896

[11] Izumi H, Kosaka N, Shimizu T, Sekine K, Ochiya T, Takase M (2013) Purification of RNA from milk whey In: Kosaka N, editor. Circulating MicroRNAs: Humana Press pp. 191–201. 10.1007/978-1-62703-453-1_15 23719952

[12] Alsaweed M, Hepworth AR, Lefèvre C, Hartmann PE, Geddes DT, Hassiotou F (2015) Human milk microRNA and total RNA differ depending on milk fractionation. Journal of Cellular Biochemistry 116: 2397–2407. 10.1002/jcb.25207 25925799 PMC5042114

[13] Nissen A, Bendixen E, Ingvartsen KL, Røntved CM (2013) Expanding the bovine milk proteome through extensive fractionation. Journal of Dairy Science 96: 7854–7866. 10.3168/jds.2013-7106 24140321

[14] Huston GE, Patton S (1990) Factors related to the formation of cytoplasmic crescents on milk fat globules. Journal of Dairy Science 73: 2061–2066. 2121808 10.3168/jds.S0022-0302(90)78885-6

[15] Izumi H, Tsuda M, Sato Y, Kosaka N, Ochiya T, Iwamoto H, et al (2015) Bovine milk exosomes contain microRNA and mRNA and are taken up by human macrophages. Journal of Dairy Science 98: 2920–2933. 10.3168/jds.2014-9076 25726110

[16] Sun J, Aswath K, Schroeder S, Lippolis J, Reinhardt T, Sonstegard T (2015) MicroRNA expression profiles of bovine milk exosomes in response to Staphylococcus aureus infection. BMC Genomics 16: 806 10.1186/s12864-015-2044-9 26475455 PMC4609085

[17] Li R, Beaudoin F, Ammah A, Bissonnette N, Benchaar C, Zhao X, et al (2015) Deep sequencing shows microRNA involvement in bovine mammary gland adaptation to diets supplemented with linseed oil or safflower oil. BMC Genomics 16: 884 10.1186/s12864-015-1965-7 26519053 PMC4628385

[18] Munch EM, Harris RA, Mohammad M, Benham AL, Pejerrey SM, Showalter L, et al (2013) Transcriptome profiling of microRNA by Next-Gen deep sequencing reveals known and novel miRNA species in the lipid fraction of human breast milk. PLoS One 8: e50564 10.1371/journal.pone.0050564 23418415 PMC3572105

[19] Yamin HB, Barnea M, Genzer Y, Chapnik N, Froy O (2014) Long-term commercial cow's milk consumption and its effects on metabolic parameters associated with obesity in young mice. Molecular Nutrition & Food Research 58: 1061–1068. 24550222 10.1002/mnfr.201300650

[20] Melnik BC, John SM, Schmitz G (2013) Milk is not just food but most likely a genetic transfection system activating mTORC1 signaling for postnatal growth. Nutrition Journal 12: 103 10.1186/1475-2891-12-103 23883112 PMC3725179

[21] Zempleni J, Baier SR, Howard KM, Cui J (2015) Gene regulation by dietary microRNAs. Canadian Journal of Physiology and Pharmacology 93: 1097–1102. 10.1139/cjpp-2014-0392 26222444 PMC4743494

[22] Baier SR, Nguyen C, Xie F, Wood JR, Zempleni J (2014) MicroRNAs are absorbed in biologically meaningful amounts from nutritionally relevant doses of cow milk and affect gene expression in peripheral blood mononuclear cells, HEK-293 kidney cell cultures, and mouse livers. The Journal of Nutrition 144: 1495–1500. 10.3945/jn.114.196436 25122645 PMC4162473

[23] Chen X, Gao C, Li H, Huang L, Sun Q, Dong Y, et al (2010) Identification and characterization of microRNAs in raw milk during different periods of lactation, commercial fluid, and powdered milk products. Cell Research 20: 1128–1137. 10.1038/cr.2010.80 20548333

[24] Howard KM, Jati Kusuma R, Baier SR, Friemel T, Markham L, Vanamala J, et al (2015) Loss of miRNAs during processing and storage of cow’s (Bos taurus) milk. Journal of Agricultural and Food Chemistry 63: 588–592. 10.1021/jf505526w 25565082 PMC4387787

[25] Cánovas A, Rincón G, Bevilacqua C, Islas-Trejo A, Brenaut P, Hovey RC, et al (2014) Comparison of five different RNA sources to examine the lactating bovine mammary gland transcriptome using RNA-Sequencing. Scientific Reports 4: 5297 10.1038/srep05297 25001089 PMC5381611

[26] Boutinaud M, Rulquin H, Keisler D, Djiane J, Jammes H (2002) Use of somatic cells from goat milk for dynamic studies of gene expression in the mammary gland. Journal of Animal Science 80: 1258–1269. 12019613 10.2527/2002.8051258x

[27] Murrieta CM, Hess BW, Scholljegerdes EJ, Engle TE, Hossner KL, Moss GE, et al (2006) Evaluation of milk somatic cells as a source of mRNA for study of lipogenesis in the mammary gland of lactating beef cows supplemented with dietary high-linoleate safflower seeds. Journal of Animal Science 84: 2399–2405. 16908643 10.2527/jas.2005-677

[28] Brenaut P, Bangera R, Bevilacqua C, Rebours E, Cebo C, Martin P (2012) Validation of RNA isolated from milk fat globules to profile mammary epithelial cell expression during lactation and transcriptional response to a bacterial infection. Journal of Dairy Science 95: 6130–6144. 10.3168/jds.2012-5604 22921620

[29] Ha M, Kim VN (2014) Regulation of microRNA biogenesis. Nature Reviews Molecular Cell Biolology 15: 509–524. 10.1038/nrm3838 25027649

[30] Vigneault F, Ter-Ovanesyan D, Alon S, Eminaga S, C Christodoulou D, Seidman J, et al. (2012) High-Throughput Multiplex Sequencing of miRNA. Current Protocols in Human Genetics: 11.12. 11–11.12. 10. 10.1002/0471142905.hg1112s73 PMC3673877 22470142

[31] Langmead B, Trapnell C, Pop M, Salzberg SL (2009) Ultrafast and memory-efficient alignment of short DNA sequences to the human genome. Genome Biology 10: R25 10.1186/gb-2009-10-3-r25 19261174 PMC2690996

[32] Friedländer MR, Chen W, Adamidi C, Maaskola J, Einspanier R, Knespel S, et al (2008) Discovering microRNAs from deep sequencing data using miRDeep. Nature Biotechnology 26: 407–415. 10.1038/nbt1394 18392026

[33] Love MI, Huber W, Anders S (2014) Moderated estimation of fold change and dispersion for RNA-seq data with DESeq2. Genome Biology 15: 550 25516281 10.1186/s13059-014-0550-8 PMC4302049

[34] Krappmann K, Weikard R, Kühn C (2012) Evaluation of a replacement method for mammary gland biopsies by comparing gene expression in udder tissue and mammary epithelial cells isolated from milk. Research in Veterinary Science 93: 970–974. 10.1016/j.rvsc.2011.12.021 22265217

[35] Lemay D, Hovey R, Hartono S, Hinde K, Smilowitz J, Ventimiglia F, et al (2013) Sequencing the transcriptome of milk production: milk trumps mammary tissue. BMC Genomics 14: 872 10.1186/1471-2164-14-872 24330573 PMC3871720

[36] Cánovas A, Rincón G, Islas-Trejo A, Jimenez-Flores R, Laubscher A, Medrano JF (2013) RNA sequencing to study gene expression and single nucleotide polymorphism variation associated with citrate content in cow milk. Journal of Dairy Science 96: 2637–2648. 10.3168/jds.2012-6213 23403202

[37] Gottesman S, Storz G (2011) Bacterial small RNA regulators: versatile roles and rapidly evolving variations. Cold Spring Harbor perspectives in biology 3: a003798 10.1101/cshperspect.a003798 20980440 PMC3225950

[38] Kosaka N, Izumi H, Sekine K, Ochiya T (2010) microRNA as a new immune-regulatory agent in breast milk. Silence 1: 7 10.1186/1758-907X-1-7 20226005 PMC2847997

[39] Jin W, Ibeagha-Awemu EM, Liang G, Beaudoin F, Zhao X, Guan LL (2014) Transcriptome microRNA profiling of bovine mammary epithelial cells challenged with Escherichia coli or Staphylococcus aureus bacteria reveals pathogen directed microRNA expression profiles. BMC Genomics 15: 181 10.1186/1471-2164-15-181 24606609 PMC4029070

[40] Bu D, Nan X, Wang F, Loor J, Wang J (2015) Identification and characterization of microRNA sequences from bovine mammary epithelial cells. Journal of Dairy Science 98: 1696–1705. 10.3168/jds.2014-8217 25622872

[41] Le Guillou S, Marthey S, Laloë D, Laubier J, Mobuchon L, Leroux C, et al (2014) Characterisation and comparison of lactating mouse and bovine mammary gland miRNomes. PLoS One 9: e91938 10.1371/journal.pone.0091938 24658750 PMC3962357

[42] Li R, Zhang C-L, Liao X-X, Chen D, Wang W-Q, Zhu Y-H, et al (2015) Transcriptome MicroRNA Profiling of Bovine Mammary Glands Infected with Staphylococcus aureus. International Fournal of Molecular Sciences 16: 4997–5013. 10.3390/ijms16034997 PMC4394461 25749476

[43] Li Z, Liu H, Jin X, Lo L, Liu J (2012) Expression profiles of microRNAs from lactating and non-lactating bovine mammary glands and identification of miRNA related to lactation. BMC genomics 13: 731 10.1186/1471-2164-13-731 23270386 PMC3551688

[44] Finucane KA, McFadden TB, Bond JP, Kennelly JJ, Zhao F-Q (2008) Onset of lactation in the bovine mammary gland: gene expression profiling indicates a strong inhibition of gene expression in cell proliferation. Functional & integrative genomics 8: 251–264. 18259788 10.1007/s10142-008-0074-y

[45] Wang M, Moisá S, Khan MJ, Wang J, Bu D, Loor JJ (2012) MicroRNA expression patterns in the bovine mammary gland are affected by stage of lactation. Journal of Dairy Science 95: 6529–6535. 10.3168/jds.2012-5748 22959945

[46] Wicik Z, Gajewska M, Majewska A, Walkiewicz D, Osińska E, Motyl T (2015) Characterization of microRNA profile in mammary tissue of dairy and beef breed heifers. Journal of Animal Breeding and Genetics 133: 31–42. 10.1111/jbg.12172 26060050

[47] Melnik BC, John SM, Schmitz G (2014) Milk: an exosomal microRNA transmitter promoting thymic regulatory T cell maturation preventing the development of atopy. Journal Translational Medicine 12: 153–161. 10.1186/1479-5876-12-43 PMC3930015 24521175

[48] Ludwig A-K, Giebel B (2012) Exosomes: small vesicles participating in intercellular communication. The international journal of biochemistry & cell biology 44: 11–15. 22024155 10.1016/j.biocel.2011.10.005

[49] Cui W, Li Q, Feng L, Ding W (2011) MiR-126-3p regulates progesterone receptors and involves development and lactation of mouse mammary gland. Molecular and Cellular Biochemistry 355: 17–25. 10.1007/s11010-011-0834-1 21526342

[50] Power ML, Schulkin J (2013) Maternal regulation of offspring development in mammals is an ancient adaptation tied to lactation. Applied & Translational Genomics 2: 55–63. 27896056 10.1016/j.atg.2013.06.001 PMC5121250

[51] Weber JA, Baxter DH, Zhang S, Huang DY, Huang KH, Lee MJ, et al (2010) The microRNA spectrum in 12 body fluids. Clinical Chemistry 56: 1733–1741. 10.1373/clinchem.2010.147405 20847327 PMC4846276

[52] Fu X, Dong B, Tian Y, Lefebvre P, Meng Z, Wang X, et al (2015) MicroRNA-26a regulates insulin sensitivity and metabolism of glucose and lipids. The Journal of Clinical Investigation 125: 2497–2509. 10.1172/JCI75438 25961460 PMC4497741

[53] McLean CS, Mielke C, Cordova JM, Langlais PR, Bowen B, Miranda D, et al (2015) Gene and microRNA expression responses to exercise; relationship with insulin sensitivity. PLoS One 10: e0127089 10.1371/journal.pone.0127089 25984722 PMC4436215

[54] Fordham JB, Naqvi AR, Nares S (2015) Regulation of miR-24, miR-30b, and miR-142-3p during macrophage and dendritic cell differentiation potentiates innate immunity. Journal of Leukocyte Biology 67: 609–613. 10.1189/jlb.1A1014-519RR PMC4501676 25990241

[55] Liu Y, Li J, Xia W, Chen C, Zhu H, Chen J, et al (2015) MiR-200b modulates the properties of human monocyte-derived dendritic cells by targeting WASF3. Life Sciences 122: 26–36. 10.1016/j.lfs.2014.11.023 25510861

[56] Pandey A, Singh P, Jauhari A, Singh T, Khan F, Pant AB, et al (2015) Critical role of the miR-200 family in regulating differentiation and proliferation of neurons. Journal of Neurochemistry 133: 640–652. 10.1111/jnc.13089 25753155

