# Supplementary figures and images for: Comparative Analysis of the miRNome of Bovine Milk Fat, Whey and Cells
Source: PLoS One. 2016 Apr 21;11(4):e0154129. doi: 10.1371/journal.pone.0154129 (PMC4839614; doi:10.1371/journal.pone.0154129)

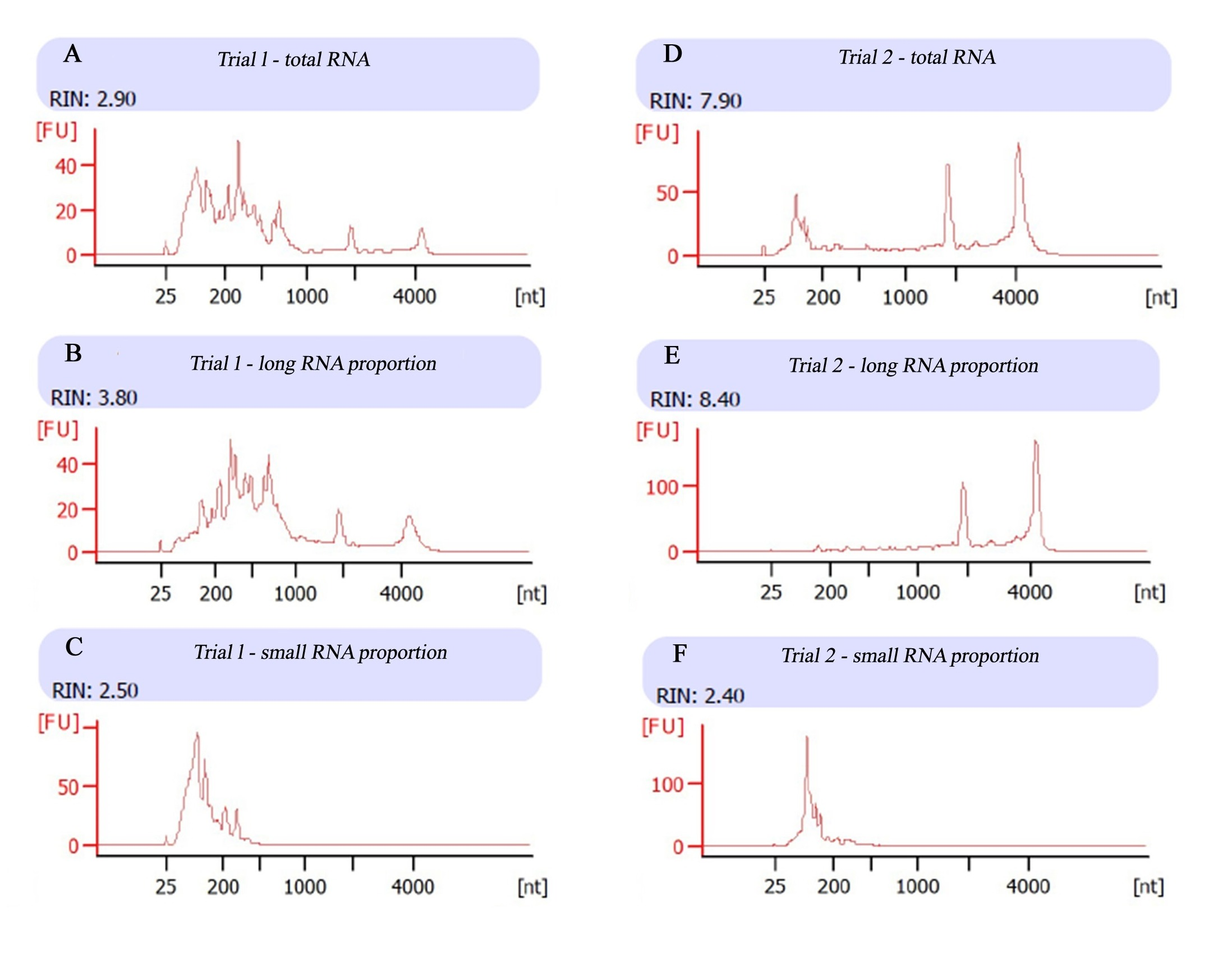

Supplement: S1 Fig — (A) Total RNA, (B) long RNA fraction and (C) small RNA fraction from trial sample 1. (D) Total RNA, (E) long RNA fraction and (F) small RNA fraction from trial sample 2. C shows that the small RNA fraction is intact despite evidence of degradation or presence of contaminating bacterial sequences (large peak from 200 to 900 nt in the electropherogram). Furthermore, improved RIN value of long RNA fraction (B and E) depended on the RIN value of the starting material (A and D). (TIF) [file pone.0154129.s001.tif]
