# Supplementary material for: Comparative Analysis of the miRNome of Bovine Milk Fat, Whey and Cells
Source: PLoS One. 2016 Apr 21;11(4):e0154129. doi: 10.1371/journal.pone.0154129 (PMC4839614; doi:10.1371/journal.pone.0154129)

S3-1 Enriched molecular functions of cell highly enriched miRNAs

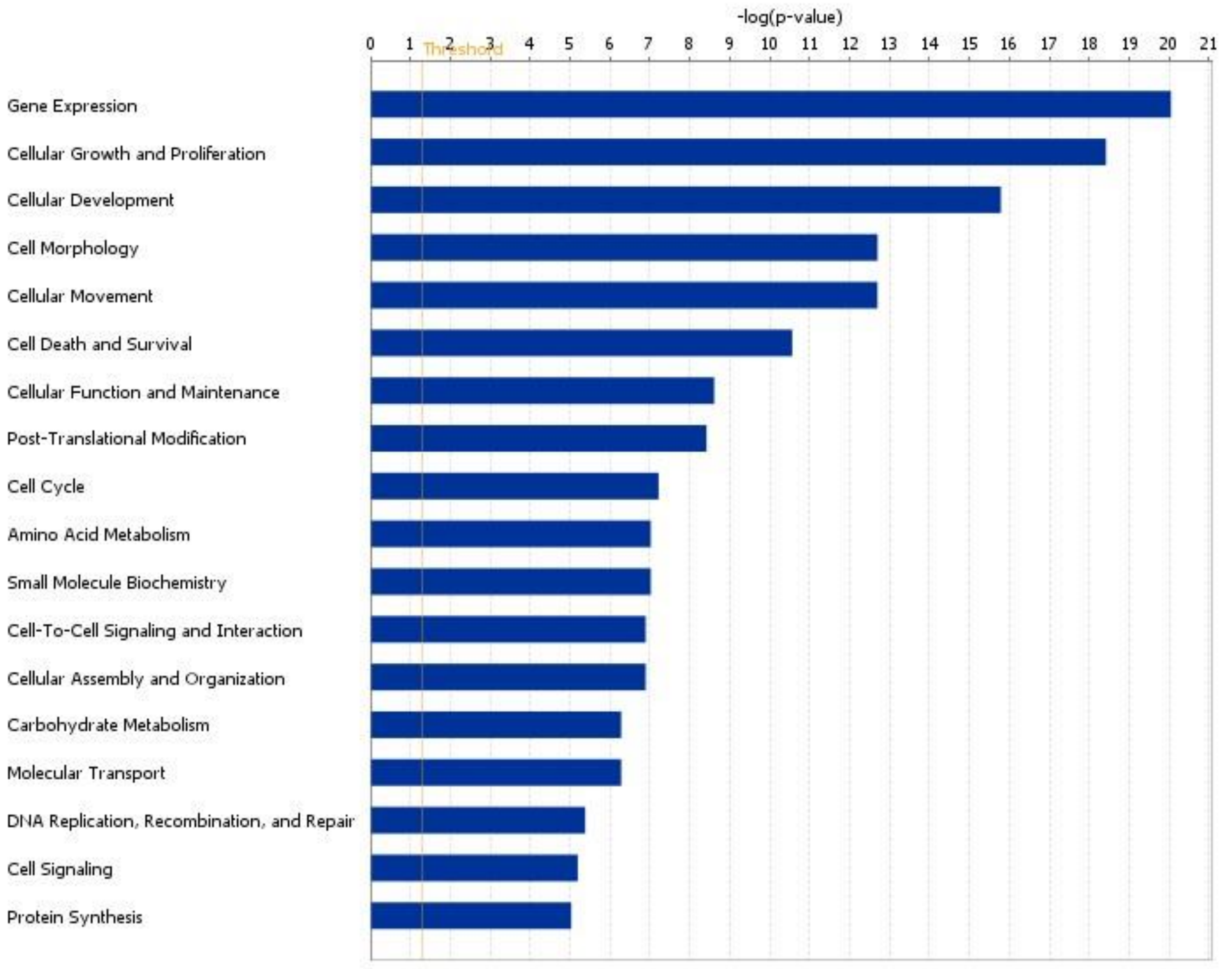

S3-2 Enriched physiological and developmental functions of cell highly enriched miRNAs

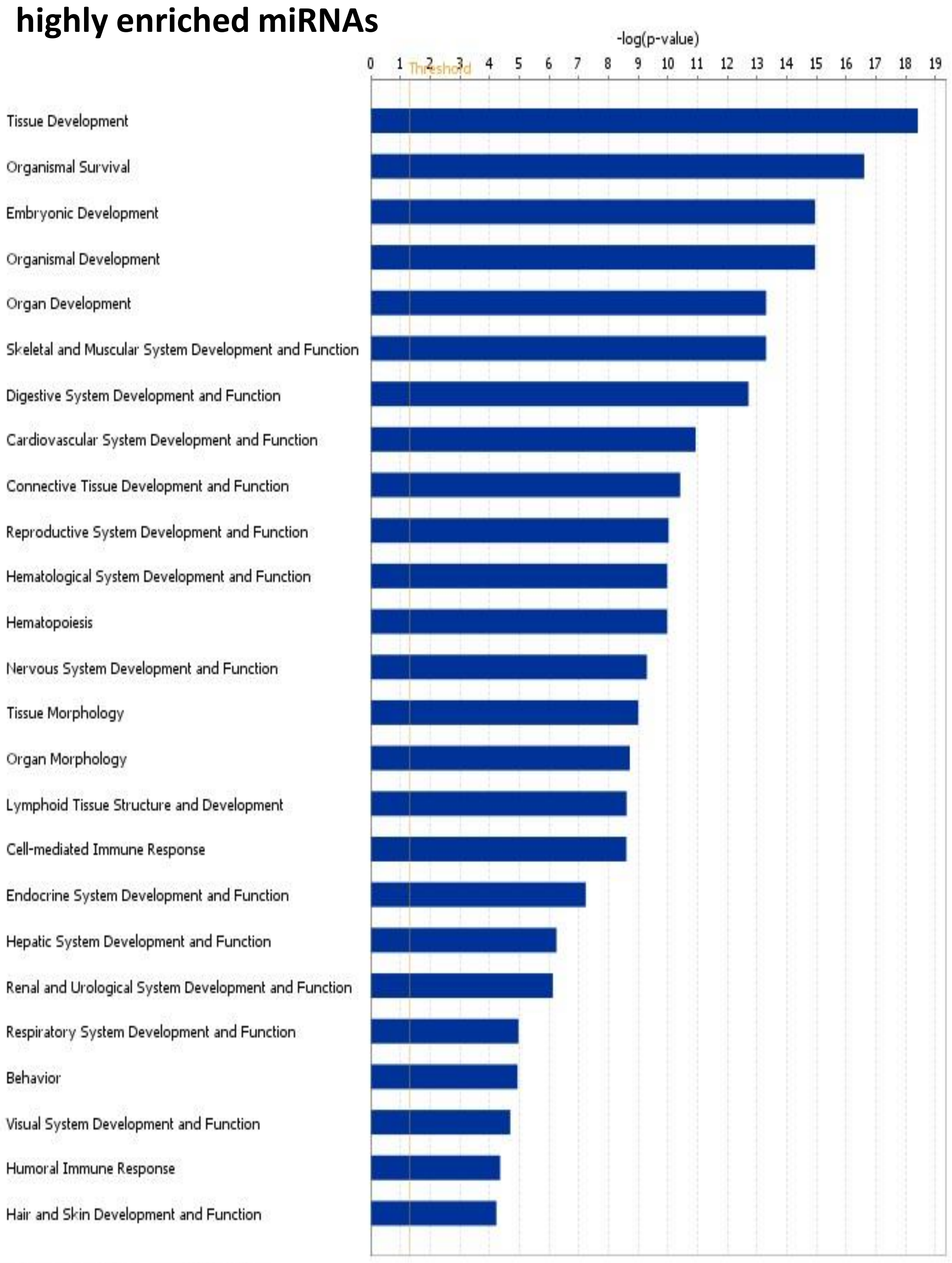

S3-3 Enriched diseases of cell highly enriched miRNAs

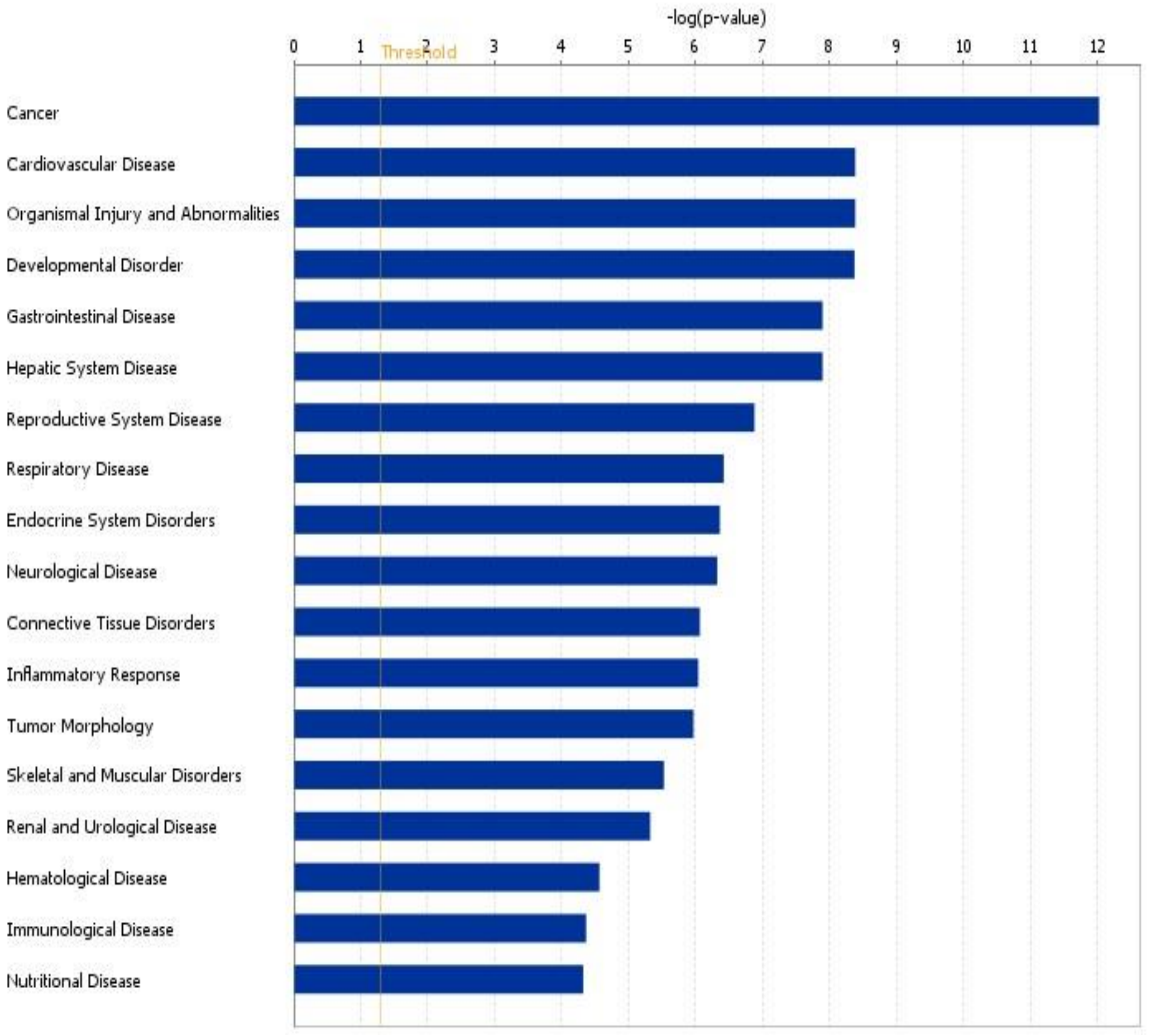

Supplement: S3 Fig — (PDF) [file pone.0154129.s003.pdf]
