# Supplementary material for: Comparative Analysis of the miRNome of Bovine Milk Fat, Whey and Cells
Source: PLoS One. 2016 Apr 21;11(4):e0154129. doi: 10.1371/journal.pone.0154129 (PMC4839614; doi:10.1371/journal.pone.0154129)

S4-1 Enriched molecular functions of mammary gland tissue highly enriched miRNAs

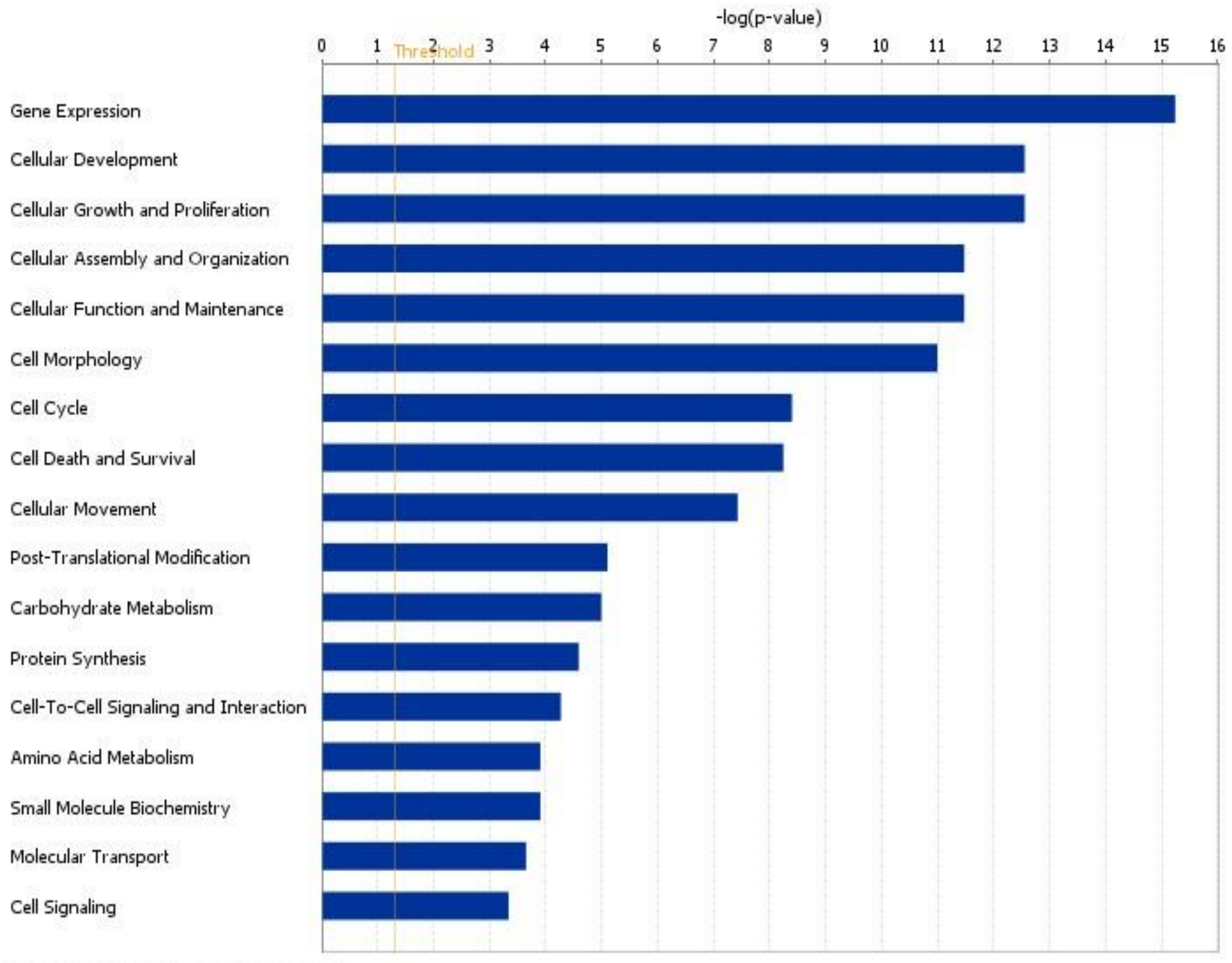

S4-2 Enriched physiological and developmental functions of mammary gland tissue highly enriched miRNAs

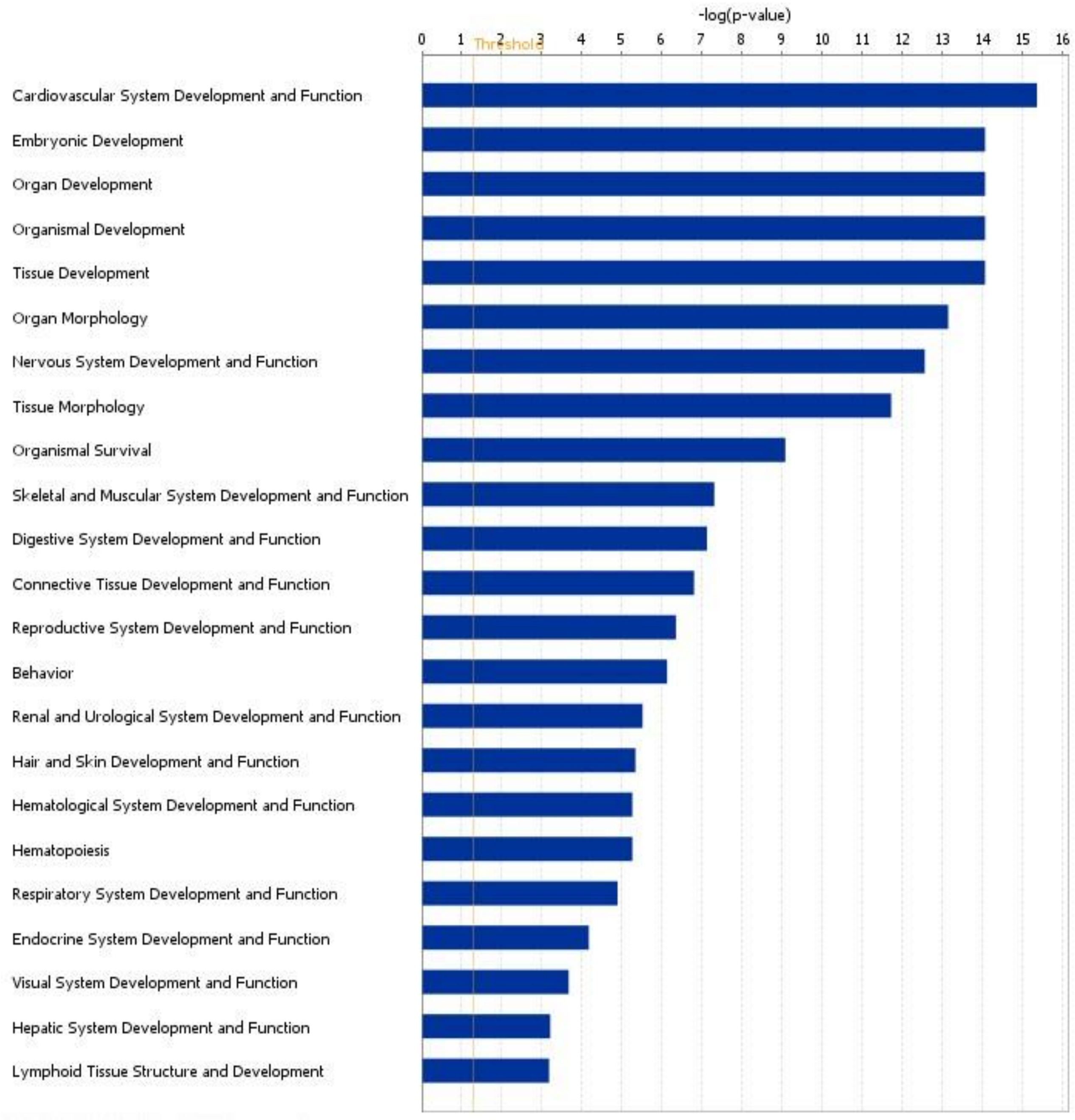

S4-3 Enriched diseases of mammary gland tissue highly enriched miRNAs

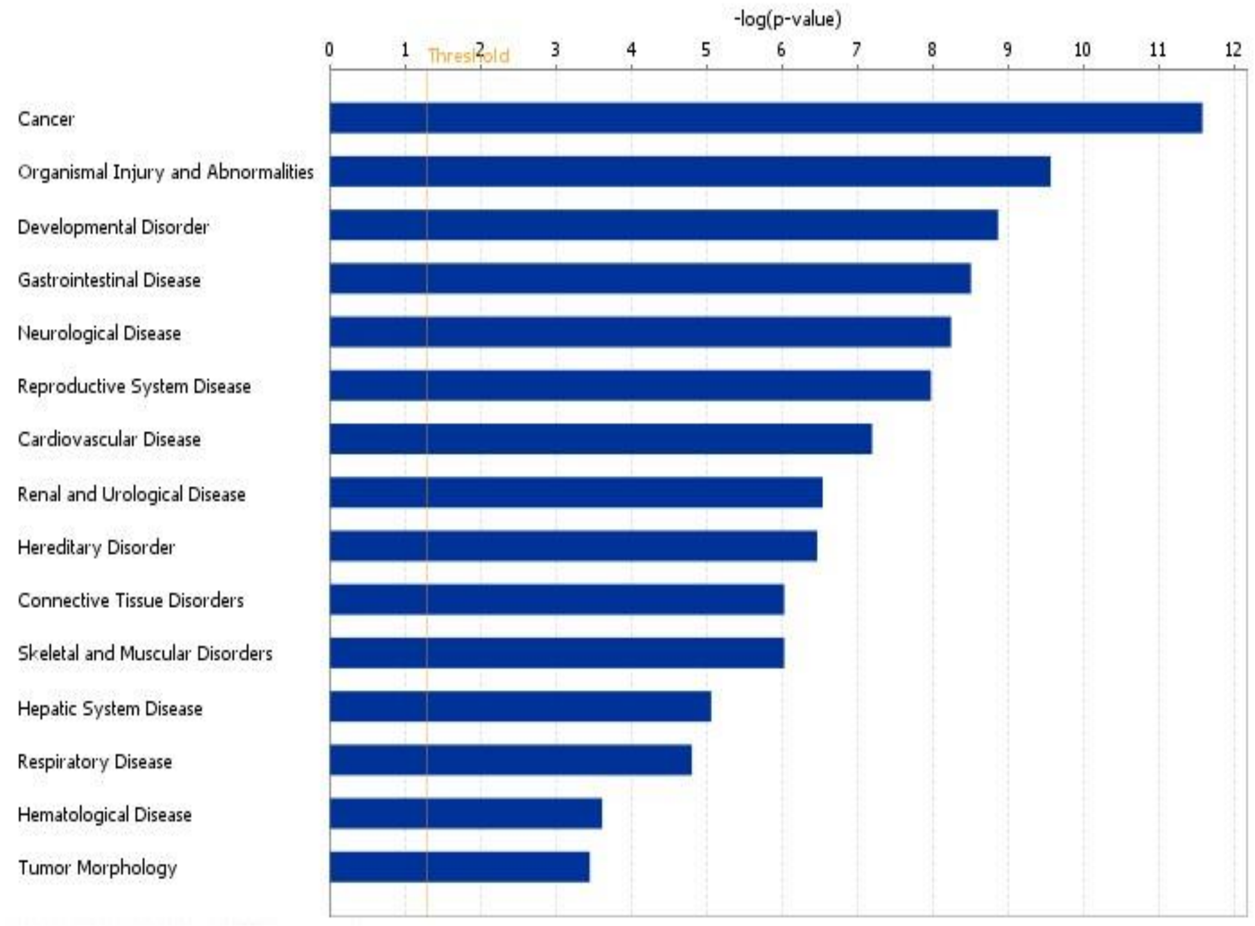

Supplement: S4 Fig — (PDF) [file pone.0154129.s004.pdf]
